# A Six Nuclear Gene Phylogeny of *Citrus* (Rutaceae) Taking into Account Hybridization and Lineage Sorting

**DOI:** 10.1371/journal.pone.0068410

**Published:** 2013-07-16

**Authors:** Chandrika Ramadugu, Bernard E. Pfeil, Manjunath L. Keremane, Richard F. Lee, Ivan J. Maureira-Butler, Mikeal L. Roose

**Affiliations:** 1 Department of Botany and Plant Sciences, University of California Riverside, Riverside, California, United States of America; 2 Commonwealth Scientific and Industrial Research Organisation Plant Industry, Canberra, Australian Capital Territory, Australia; 3 DBES, Gothenburg University, Gothenburg, Sweden; 4 United States Department of Agriculture–Agricultural Research Service National Clonal Germplasm Repository for Citrus and Dates, Riverside, California, United States of America; 5 Agriaquaculture Nutritional Genomic Center, Centro de Genómica Nutricional Agroacuícola, Genomics and Bioinformatics Unit, Temuco, Chile; Virginia Tech, United States of America

## Abstract

**Background:**

Genus *Citrus* (Rutaceae) comprises many important cultivated species that generally hybridize easily. Phylogenetic study of a group showing extensive hybridization is challenging. Since the genus *Citrus* has diverged recently (4–12 Ma), incomplete lineage sorting of ancestral polymorphisms is also likely to cause discrepancies among genes in phylogenetic inferences. Incongruence of gene trees is observed and it is essential to unravel the processes that cause inconsistencies in order to understand the phylogenetic relationships among the species.

**Methodology and Principal Findings:**

(1) We generated phylogenetic trees using haplotype sequences of six low copy nuclear genes. (2) Published simple sequence repeat data were re-analyzed to study population structure and the results were compared with the phylogenetic trees constructed using sequence data and coalescence simulations. (3) To distinguish between hybridization and incomplete lineage sorting, we developed and utilized a coalescence simulation approach. In other studies, species trees have been inferred despite the possibility of hybridization having occurred and used to generate null distributions of the effect of lineage sorting alone (by coalescent simulation). Since this is problematic, we instead generate these distributions directly from observed gene trees. Of the six trees generated, we used the most resolved three to detect hybrids. We found that 11 of 33 samples appear to be affected by historical hybridization. Analysis of the remaining three genes supported the conclusions from the hybrid detection test.

**Conclusions:**

We have identified or confirmed probable hybrid origins for several *Citrus* cultivars using three different approaches–gene phylogenies, population structure analysis and coalescence simulation. Hybridization and incomplete lineage sorting were identified primarily based on differences among gene phylogenies with reference to null expectations via coalescence simulations. We conclude that identifying hybridization as a frequent cause of incongruence among gene trees is critical to correctly infer the phylogeny among species of *Citrus*.

## Introduction

The genus *Citrus* L. includes commercially important cultivars grown in tropical to temperate parts of the world over several thousands of years [1]. *Citrus* and its relatives are native to Southern to Eastern Asia, Malesia, New Caledonia and Australia [1]. Citrus biology is complicated due to extensive hybridization, polyembryony and vegetative methods of propagation. Taxonomic uncertainty has resulted in different treatments of the diversity within the main cultivars of citrus and of what constitutes the genus. Under some taxonomies, only three wild species (or “basic species”) are recognized among cultivated Asian *Citrus*: citron (*Citrus medica* L.), mandarin (*C. reticulata* Blanco) and pummelo (*C. maxima* (Burm.) Merr.) – the remainder being derived by hybridization [2], [3], [4]. The most widely accepted classification of *Citrus* and closely related genera was proposed by Swingle and Reece [1] who recognized 16 species in the genus *Citrus*, or, 29 species when other taxa of Group C are included in the genus (see below). In contrast, another *Citrus* taxonomist, Tanaka, has identified as many as 162 species in the genus [5].

Swingle and Reece’s [1] classification recognizes three groups in the sub tribe Citrinae based on fruit characteristics: Group A, the “Primitive Citrus Fruit Trees”; Group B, the “Near-Citrus Fruit Trees”; and Group C, the “True Citrus Fruit Trees”. Group C includes the genus *Citrus* along with five other mostly cross-compatible genera: *Clymenia* Swingle [6], *Fortunella* Swingle [7], *Eremocitrus* Swingle [8], *Microcitrus* Swingle [7], [9] and *Poncirus* Raf. [1]. Genera belonging to the “true citrus fruit trees” have been studied extensively; phylogenetic analysis of chloroplast sequence data clusters them in a single clade designated by Bayer et al. as *Citrus* s.l. [10], [11].

Both man-made and natural hybrids of *Citrus* L. (Rutaceae) have been cultivated for centuries [1]. The genus *Citrus* and its near relatives (Group C) have probably diverged recently, estimated to be c. 4–12 million years ago [12], [13]. Therefore, hybridization and lineage sorting are likely to be the major challenges for the inference of species phylogeny in *Citrus*. Intergeneric sexual hybridization between *Citrus* (*sensu stricto*) and these other closely related genera has resulted in new hybrids in breeding programs [14]. *Citrus* is mainly propagated by grafting in order to retain the desirable horticultural characters. Such practices, in addition to the natural adventitious nucellar embryony seen commonly in sweet oranges, mandarins and grapefruit, play an important role in stabilizing and perpetuating hybrids that might otherwise be eliminated in nature.

Earlier work on the phylogenetic relationships of *Citrus* cultivars and their close relatives used morphological comparisons [1], chloroplast sequences or markers (cpDNA) [15], [16], [17], [18], mitochondrial sequences or markers [19], [20], restriction fragment length polymorphisms (RFLPs), random amplified polymorphic DNA markers (RAPDs) [21], [22], isozymes [23], sequence characterized amplified region (SCAR), and simple sequence repeat markers (SSRs) [24]. The most comprehensive study of citrus phylogeny used sequences of nine cpDNA loci [10]. In *Citrus*, the cpDNA was presumed to have a strictly maternal inheritance pattern [16], and studies using cpDNA were assumed to be informative about the female ancestry of hybrids. However, recent work [25] using Chandler pummelo X Fortune mandarin indicated that organelles may also be inherited from the male parent. Phylogenetic inferences derived from analysis of nuclear gene sequences may be more informative in determining the relationships between the different cultivars.

In *Citrus*, phylogenetic analysis using nuclear gene sequences has so far been limited to single nucleotide polymorphisms (SNPs) derived from randomly sampled regions [26]. In many studies, sequence data has been concatenated before analysis and this may mislead phylogenetic inference when divergent signals are present [27], [28]. It is also conceivable that certain hybrids contain alleles that cluster in different clades of a phylogenetic tree. Previous analysis of the hybrid taxa with a single terminal depicted in the phylogenetic tree for each individual may not address heterozygosity adequately [26]. Thus, the inferences of citrus phylogeny have been limited by analysis of single gene markers, concatenation of multiple sequences in scattered genomic locations and depiction of single alleles for hybrid accessions; all three approaches are major drawbacks for a group known to contain hybrids and likely to be affected by lineage sorting.

Hybridization and (incomplete) lineage sorting cause inconsistencies in phylogenetic studies. Detection of incongruence has been facilitated by many new tests [29] and methods [30] but often, it has been difficult to untangle the processes that cause incongruence. The common causes of such inconsistencies between gene trees are known to be due to selection, recombination, gene duplication (paralogy), or, during phylogenetic analysis, choice of incorrect nucleotide substitution model or, minor technical discrepancies [31]. If these are ruled out, hybridization and the incomplete sorting of ancestral polymorphisms between speciation events (hereafter referred to as lineage sorting) often remain as viable hypotheses to explain incongruence [32], [33], [34], [35], [36], [37].

In the present study, we inferred the phylogeny of *Citrus* using sequence from six nuclear loci, of which only one pair was significantly linked. Haplotype sequences were generated and analyzed to understand the genetic composition of the hybrid accessions. We also analyzed multi-locus simple sequence repeat data and compared the inferred population structure with the current phylogenetic analysis. As conflicting phylogenies were found for different loci, we untangled the possible causes of these conflicts via coalescent simulations. This is based on the principle that hybrids can contain alleles drawn from very different parts of the tree in different genes. But simulations under the coalescent place limits on how probable different placements of alleles can be in different genes without hybridization, thus providing a null expectation. The conflicting phylogenies observed for different loci were explained using a comparison of the trees generated by coalescence (under various assumptions) and the original gene trees. Our study uses a method to identify hybridization and lineage sorting [13], [37], [38], the two important factors in understanding citrus phylogeny. We made phylogenetic inferences for the accessions included in the study based on a combination of phylogenetic tree data, STRUCTURE analysis and coalescent simulation data.

## Materials and Methods

### Sampled Taxa, DNA Extractions, Amplification and Sequencing

Taxa used for the study were obtained from the Citrus Variety Collection, Riverside, CA. Of 33 accessions selected (Table 1), 25 were cultivars of *Citrus*, two of *Poncirus* and one accession each of *Fortunella*, *Microcitrus, Naringi*, *Citropsis*, *Atalantia* and *Swinglea*. DNA was extracted from fresh or silica gel dried leaves using a modified Cetyl trimethyl ammonium bromide (CTAB) method [13], [39] or using DNAzol (Molecular Research Center, Cincinnati, OH) as per the manufacturer’s protocol. Briefly, 1 g of leaf was pulverized in 4 ml of 100 mM Tris HCl, pH 8.0 with 2% CTAB, 1.4 M NaCl, 20 mM EDTA, 2% PVP 40, 0.2% β mercaptoethanol (added just before extraction) and 40 µg of RNase A, incubated at 65^0^ C for 30 min, then centrifuged. The supernatant was extracted twice with chloroform: iso-amyl alcohol (24∶1) and ethanol precipitated.

**Table 1:** Study taxa and voucher information.

Cultivar name	Abbr	CRC#	Swingle name	Tanaka name
‘Arizona 861 S-1′ citron*	ARZ	3878	*Citrus medica* L.	
Indian sour citron (Zamburi)*	IND	661	*C. medica* L.	
‘South Coast Field Station’ citron	SCF	3546	*C. medica* L.	
Mountain citron*	HLM	3780	*C. halimii* B.C. Stone	
‘Kalpi’ lime	KLP	1455	*C. aurantifolia* (Christm.) Swing.	*C. webberi* Wester
‘Winged’ lime (Tamisan, Talamisan)	WGL	2320	*C. aurantifolia* (Christm.) Swing.	*C. longispina* Wester
‘Mexican’ lime*	MEX	3822	*C. aurantifolia* (Christm.) Swing.	
‘Palestine’ sweet lime*	PAL	1482	*C. aurantifolia* (Christm.) Swing.	*C. limettioides* Tanaka
‘Frost Eureka’ lemon*	FRS	3005	*C. limon* (L.) Burm.f.	
‘Frost Owari’ satsuma	FOW	3178	*C. reticulata* Blanco	*C. unshiu* Marc.
‘Nasnaran’ mandarin*	AMB	2485	*C. reticulata* Blanco	*C. amblycarpa* Ochse.
‘Tien Chieh’ mandarin*	TNC	2590	*C. reticulata* Blanco	
‘Scarlet Emperor’ mandarin	SEM	3326	*C. reticulata* Blanco	
‘Encore’ mandarin	ENC	3569	*C. reticulata* Blanco	
‘Korai’ mandarin	NIP	3228	*C. reticulata* Blanco	*C. nippokoreana* Tanaka
‘King’ mandarin*	KNG	3845	*C. reticulata* Blanco	*C. nobilis* Lour.
‘Cleopatra’ mandarin*	CLE	3844	*C. reticulata* Blanco	*C. reshni* Hort. ex Tanaka
‘Bouquet de Fleurs’ sour orange*	BDF	571	*C. aurantium* L.	
‘Rubidoux’ sour orange*	RBD	3855	*C. aurantium* L.	
‘Washington’ navel orange	WNO	1241	*C. sinensis* (L.) Osbeck	
‘Kao Pan’ pummelo	KPN	2242	*C. maxima* (Burm.) Merrill	
‘Kao Panne’ pummelo	KPE	2248	*C. maxima* (Burm.) Merrill	
‘Mato Buntan’ pummelo	MTB	3945	*C. maxima* (Burm.) Merrill	
‘Ichang’ papeda	ICH	2327	*C. ichangensis* Swing.	
‘Hanayu’ papeda	HNU	3469	n/a	*C. hanaju* Siebold
‘Flying Dragon’ trifoliate	FDR	3330	*Poncirus. trifoliata* (L.) Rafinesque	
‘Pomeroy’ trifoliate	PMY	1717	*P. trifoliata* (L.) Rafinesque	
‘Nagami’ kumquat	NGM	3877	*Fortunella margarita* Swingle	
Finger lime (common name)	MIC	3672	*Microcitrus australasica* Swingle	
n/a		3287	*Atalantia ceylanica* (Am.) Oliv.	
n/a		2879	*Naringi crenulata* (Roxb.) Nicolson	
n/a		4043	*Swinglea glutinosa* Merr.	
n/a		3286	*Citropsis gabunensis* Swingle & Kellerman	

Abbreviations listed are used in some supplemental figures and tables. The species designations used by Swingle and Tanaka are shown (Tanaka names omitted where they are the same as the Swingle name to the left). CRC# number refers to identification number of accessions maintained at the Citrus Variety Collection, Riverside, California. Asterisk (*) indicates accessions with about 10% or higher level of admixture of different groups of citrus as analyzed in the present study utilizing SSR markers (Table S6).

Polymerase chain reaction (PCR) amplification was carried out using primers listed in Table 2 for six loci: malate dehydrogenase (MDH), P12 blight related gene (P12), aspartate transcarbamylase (ATC), limonoid glucosyltransferase (LGT), an NBS-LRR resistance gene in the CTV resistance region of citrus (CTV11) and beta-carotene hydroxylase gene (HYB). Primers were designed from unigenes in the citrus EST database C_38 relaxed assembly (http://www.harvEST.org) or from published sequences [40]. Each PCR comprised 20mM Tris-HCl, pH 8.8 with 10 mM KCl, 10 mM (NH_4_)_2_SO_4_, 2 mM MgSO_4_, 0.1% Triton X-100 and either 0.05 or 0.025 units/µl of Taq DNA polymerase (NEB) or HotStar polymerase (QIAGEN), respectively. Thermal cycling was done using: 1×94^0^ C [3′], 34×(94^0^ C [15–30″], 50–56^0^ C [15–45″], 72^0^ C [30–60″]), depending on the region. Products were purified, then sequenced at the University of California, Riverside (Sanger sequencing using Applied Biosystems® 3730×l DNA sequencer), or at the Australian National University, Canberra (Sanger sequencing using Applied Biosystems® 3730 DNA sequencer). Products from selected samples (of MDH, P12, ATC, LGT and CTV11) observed to be heterozygous in direct sequencing were cloned in pSC-A (Stratagene). All products from HYB were cloned in pGEM-T-easy (Promega). Sequencing of all clones was done using vector-based primers. Sequences from *Naringi*, *Citropsis*, *Atalantia* and *Swinglea* served as out groups in the analysis.

**Table 2:** Primers used to amplify gene fragments targeted for sequence analysis.

Locus	Primer	Primer Sequence	Length	*Citrus*Unigene
ATC	Cit 199	CATTTGTACCAGCAAGCGAAG	1028	18286
	Cit 200	TCCCAACTTGACTCTGAATGC		
CTV 11	Cit 244	GTCAATTCTTCTAGACATG	987	36980
	Cit 245	GCCAGATGTCATAGAATG		
HYB - initial	F 120	CTGCCGTCATGTCTAGTTTTGG	c. 1300	
	R 618	ACACCGTCGAATTTATCCGAGT		
HYB - final	F 154	GGCTGTCATGGCTGTTTATTACA	c. 1200	
	R 613	GTCGAATTTATCCGTAGTGGTGAA		
LGT	Cit 226	ATGGGAACTGAATCCCTT	881	2650
	Cit 243	TCTTCAACTTGTTCTTGC		
MDH	2002 F1	GCTCCTGTGGAAGAGACCC	995	3400
	2002 R1	GCTCCAGAGATGACCAAAC		
P12	Cit 203	ACGAGAGCCATTAGCCGTAG	996	787
	Cit 204	TCGCCGTACATAGCGATCC		

Citrus unigene numbers correspond to unigenes listed in the EST database, harvEST Citrus, assembly C_38 (http://www.harvEST.org).

At least eight overlapping cloned or direct sequences were generated for all samples. Comparisons between direct sequences and clones were also made. Direct sequences were examined using Phred/Phrap/Consed trace file analysis and sequence assembly software [41], [42]. Single nucleotide polymorphisms were identified using the Polyphred tool in the Phred/Phrap/Consed package. Regions with multiple peaks in a single position were scored as heterozygous and haplotypes were deciphered either using cloned sequences or PHASE analysis (v. 2.1) [43]. Many direct sequences had heterozygous insertions and deletions (indels) that were inferred using the Mutation Surveyor program (Soft Genetics®) or by inspection of sequences from clones. Unique clones differing by one or two nucleotides from others were attributed to possible PCR error and not included as novel alleles. Automated alignments of haplotypes were made using Clustal X [44] with minor manual adjustments. Sequences generated were deposited in GenBank (accession numbers: EU254083–254133 [MDH]; EU254033–254082 [P12]; EU253980–254032 [ATC]; EU254173–254216 [LGT]; EU254134–254172 [CTV11], GQ892192–GQ892246 [HYB]). Chloroplast sequence analysis was conducted using publicly available sequences from GenBank.

Possible linkage of the six genes analyzed was determined by BLAST searches against an unreleased draft version (1.0) of the Clementine mandarin reference genome sequence (phytozome.org) composed of near whole chromosome scaffolds (Roose: personal communication). Each sequence had a single hit of 94% or higher identity.

### Data Analysis

Recombination was tested within the six loci for in-group sequences (*Citrus*, *Fortunella*, *Microcitrus*, *Poncirus*) using RDP3 version 3.15 [45] with the following subroutines: RDP [46], Chimaera and MaxChi [47], [48], 3Seq [49] and Bootscan [50], [51]. A window size of 200 and step size of 20 was used for the Bootscan analysis. Bonferroni correction for multiple testing was included.

Since most *Citrus* accessions are known to be hybrids, we generated two haplotype sequences for each sample and analyzed them separately for each included gene. The direct PCR sequences and clone sequences were used to generate the haplotype sequence data. Sequences obtained from the six gene fragments were analyzed by both maximum parsimony and Bayesian methods. Phylogenetic analysis was performed by maximum parsimony (MP) using PAUP* version 4.10 b [52] and Bayesian analysis (BA) in MrBayes version 3.1.2 [53]. Two Markov Chain Monte Carlo (MCMC) runs, each with ten chains, were done for 5 M generations and sampled every 5 K generations. Although four chains are the default, additional chains will increase the chance that the analysis will not be trapped by a solution that is locally (not globally) optimal. For BA, we used default priors for the topology (uniform setting), branch lengths (unconstrained), the four stationary frequencies of the nucleotides and the six different nucleotide substitution rates (flat Dirichlet, all values are 1.0), the proportion of invariable sites (uniform distribution between 0 and 1) and the shape parameter of the gamma distribution of rate variation (uniform distribution between 0 and 1). The state frequencies of the indel characters were scored using nucleotides (to use the same matrix for MP). The state frequency priors for indels were empirically derived and the “coding =  variable” command used for this partition. About 25% of the trees were discarded as “burn-in”. The data was partitioned into exons, introns and indels (insertions and deletions) and analyzed by varying the partition models (Table S1). Models were evaluated using Bayes factors (posterior odds of one hypothesis when the prior probabilities of the two hypotheses are equal) [54]. Standard deviation of split frequencies generally reached 0.01 (Table S1) and convergence in topology and relative branch lengths among models was observed. However, absolute branch lengths did not always converge, suggesting that these are sometimes overestimated [55]. Chronograms representing time durations along branch lengths were constructed from the phylogenetic data using r8s program (http://loco.biosci.arizona.edu/r8s/), with independent calibration, thus avoiding potential error caused by the lack of convergence in absolute branch length.

### STRUCTURE Analysis

In order to compare a more genome-wide population admixture signal in the accessions studied, we conducted a multi locus genotype data analysis to investigate population structure. We used the program STRUCTURE 2.3 [56] to infer population structure of a subset of accessions (212 individuals) using data from 24 nuclear simple sequence repeats (SSR) previously published by Barkley et al [24]. Accessions were selected to reduce the number of known interspecific hybrids in the data and most taxa used for gene sequencing and SNP discovery were included (Table S5). We used the admixture model with the option that “allele frequencies are correlated” among populations. Multiple runs were performed with 1 million generations, after 500,000 generations were discarded as “burnin”. The number of populations analyzed varied from 1–10 (designated by K value) with the allele frequency prior, lambda, set to 0.62. The recommended probability value, Pr (X/K) in this analysis should be a negative number close to zero [56]. We obtained this value when K was set to 6. At K = 6, the value of the Dirichlet parameter for calculating the degree of admixture, alpha, varied between 0.035 to 0.040. The mean value of alpha was 0.0359. The smallest value of K that captured the major structure of the data was selected [56]. The results were stable and consistent when the number of populations designated by K was selected as 6.

### Coalescence Simulation Test

In phylogenetic studies, coalescence simulation is a method that predicts the time it takes for all different alleles in a population to coalesce to the most recent common ancestor (MRCA). Such simulations are done under various assumptions. These tests are useful to study the validity of different hypotheses used to explain phylogenetic patterns observed.

We simulated under the coalescent [13], [37], [38] using the hypothesis that lineage sorting alone causes the differences that are observed between gene trees. In brief, the variation that could arise by lineage sorting alone was simulated using the coalescent for each gene tree (treating each gene tree as a surrogate for the unknown species tree). Tree to tree distances were calculated between the simulated trees and the original source gene trees. If the hypothesis that lineage sorting alone has caused the difference between the trees is true, the simulated trees and the original trees will be similar. If the distances between the simulated trees (assuming lineage sorting as the only option) and the original trees are significantly different, other processes are likely to be involved. In this situation, we reject the null hypothesis that lineage sorting alone has generated the observed gene trees and consider other processes. The expectation for how different two gene trees need to be to reject the null (the critical value hereafter) has been established by simulation using known species trees [38] (Pfeil, unpublished).

For the simulation test, we drew 20 trees from the posterior distribution for each gene (Figures S1A, S1B and S1C) and calculated all tree to tree distances from this pool of trees [13], [37] (rather than from the consensus). We also evaluated the critical value used when calculating the test statistic (φ hereafter) for three genes and found that a critical value of 80% yielded <5% type 1 error rate on the same example tree (type I error is committed if we reject the null hypothesis when it is true; see Appendix S1).

We sequentially removed individuals from all trees and re-evaluated φ until we found the smallest set of individuals that resulted in φ ≤0 (thus the null hypothesis of lineage sorting alone – to explain differences in the observed gene trees – can no longer be rejected). We started by removing each individual alone to determine those with the greatest effect on φ (data not shown). We then removed groups of these individuals until φ ≤0. Until φ ≤0, hybrids remain in the data set. We also used 10 random sets of individuals to see if our selected set comprised the most likely hybrids. Further details and a step by step implementation of the method followed are described in the Appendix.

### Species Tree Estimation

After the hybrid individuals were removed (see Results), we determined the species phylogeny of remaining non-hybrid individuals under the assumption that only lineage sorting of ancestral polymorphisms is the cause of incongruence among gene trees. We used the quick “Deep Coalescence Multiple Loci” module in Mesquite program to measure discordance between a gene tree and a species tree [57]. The “Deep Coalescence Multiple Loci” module infers a species tree by accommodating gene tree differences by minimizing the number of assumed lineage sorting events (where the only process that is assumed to cause gene tree differences is lineage sorting, i.e., deep coalescence). We used the supported topology alone (PP≥0.95) based on BA consensus trees (alleles sampled as per the coalescence test), did not auto-resolve polytomies and kept the root as per our outgroup-rooted analyses. The three loci used for the hybridization detection (MDH, LGT and HyB) were analyzed initially.

We continued the analysis by serially including P12 (four locus analysis), chloroplast relationships (five locus analysis) and CTV11 (six locus analysis). The individuals used in the chloroplast study were not the same as used in the nuclear genes studied here but grouped into species clusters consistent with previous results [24] in most cases. Since CTV11 sequences were considered to have recombination based on RDP3 analysis, we constructed CTV11 trees using only the fragment (nucleotides 1–295) that did not show recombination (Figure S2).

Since CTV11 might also be affected by paralogy (see Results), we examined an approximate measure of the rate of change by calculating the distance between Washington navel orange and trifoliate orange accessions in all genes as well as between pummelo and trifoliate orange in CTV11 using DNADIST in BioEdit (Kimura-2-parameter method; see Discussion). Only the first 295 nucleotides of CTV11 were used, as above, because a mixture of characters that have different histories can affect molecular date estimations [58]. “Deep Coalescence Multiple Loci”, based only on topology, should accommodate paralogy as well as lineage sorting and therefore it may be appropriate to include CTV11 in the species tree inference. We report the results obtained including CTV11 and also without this gene.

## Results

### Sequence Data Analysis

**Figure 1:**
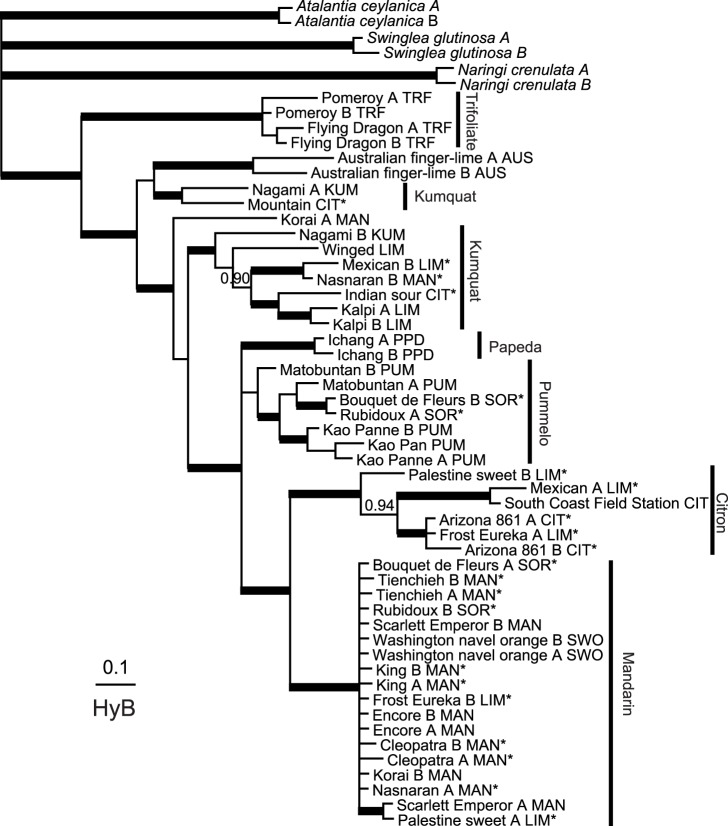
Bayesian consensus phylogram of HYB sequences from *Citrus* and related genera. Clades with posterior probabilities (PP) of 0.95–1.00 are marked with bold branches; clades with PP less than 0.95 but at least 0.90 are shown above or to the left of branches. Accessions belonging to *Citrus, Poncirus, Fortunella* and *Microcitrus* are indicated by cultivar or common names with details in Table 1. Latin names are used for outgroup genera only. Suffix A and B refer to the two haplotypes. Accessions without a suffix have only one haplotype. The traditional cultivar group to which the accession was previously assigned is indicated by three letter abbreviation following the cultivar/haplotype information. The abbreviations used are: CIT: citron; MAN: mandarin; PUM: pummelo; TRF: trifoliate orange; SWO: sweet orange; KUM: kumquat; SOR: sour orange; PPD: papeda; LIM: lime and lemon. Groups of alleles discussed in the text are marked by cultivar grouping names on the right. Cultivars that indicated an admixture of more than 10% (Table S6) are marked by an asterisk. Scale bar for branch lengths represents substitutions per site.

**Figure 2:**
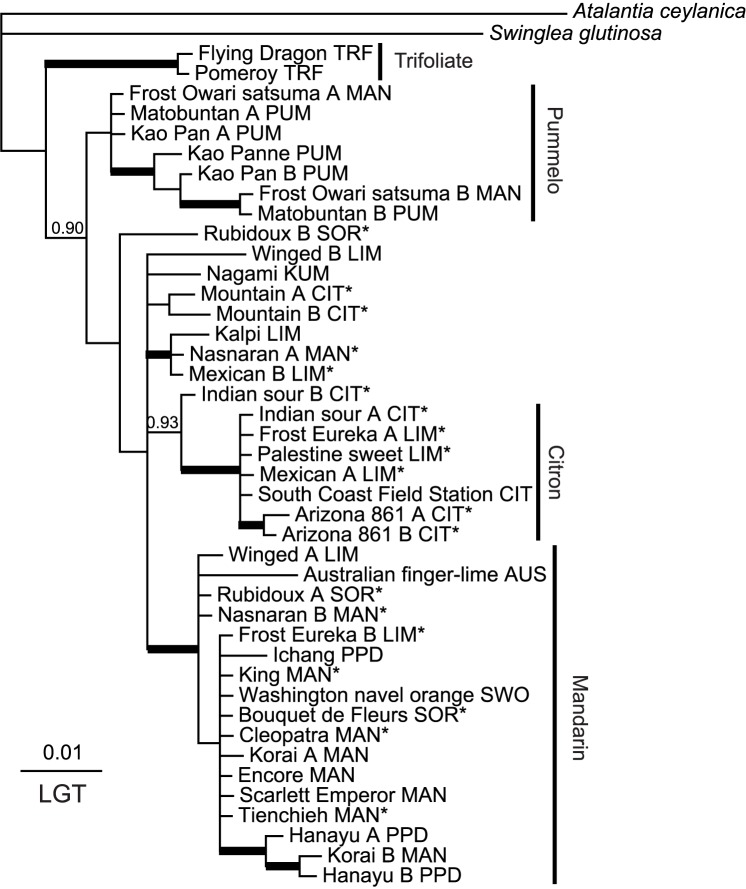
Bayesian consensus phylogram of LGT sequences from *Citrus* and related genera. Details as in Figure 1.

**Figure 3:**
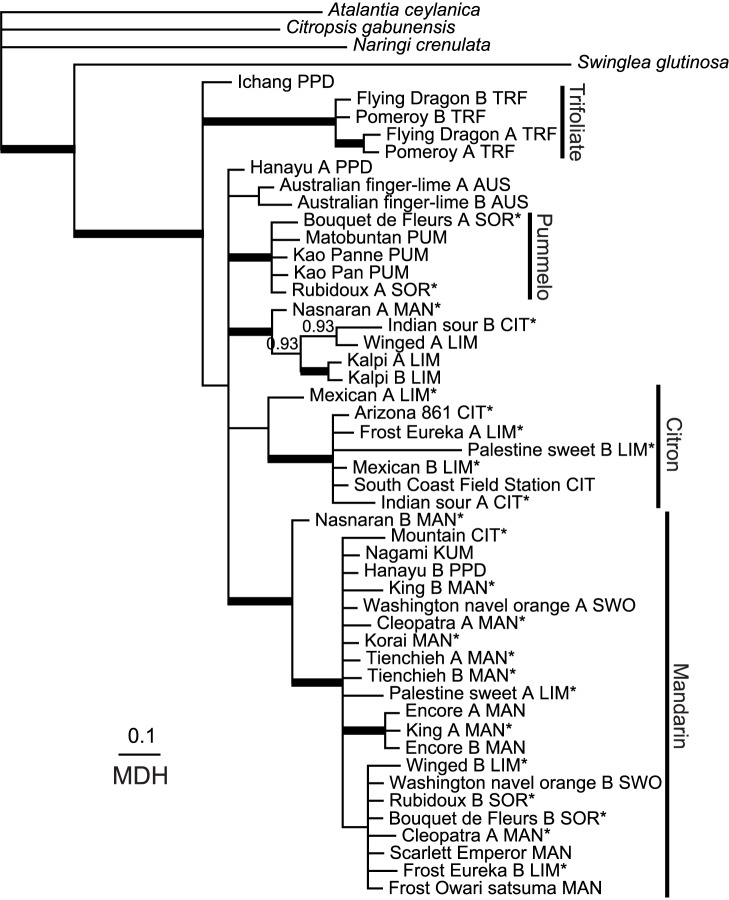
Bayesian consensus phylogram of MDH sequences from *Citrus* and related genera. Details as in Figure 1.

A total of 5632 characters from six genes were analyzed by parsimony. There were 474 parsimony-informative characters with an overall homoplasy index ranging from 0.128 to 0.461. The trees generated from ATC sequences showed high homoplasy (HI = 0.46) and poor bootstrap support for most groups, and hence were not very useful (Table S3; trees not shown). Phylogenetic trees constructed based on the best-fitting models for each gene using BA show these general patterns: i) citrons form a well supported clade in 5 gene trees; ii) mandarin are in a clade with strong support in 4 gene trees; iii) most pummelos cluster together in 5 gene trees and iv) the trifoliates clustered well in all 6 gene trees (Figures 1, 2, and 3, Figures S2, S4 and S5). The origins of the alleles in each accession inferred by the results of BA and parsimony data indicated that Arizona citron, South Coast Field Station citron, Scarlett Emperor mandarin, Encore mandarin, Kao Pan pummelo, Flying Dragon trifoliate and Pomeroy trifoliate had only alleles of citron, mandarin, pummelo or trifoliate as expected. All cultivars showed heterozygosity except South Coast Field Station citron (Table S2). We were able to see general clustering patterns across the four genes showing reasonable resolution and lacking recombination (i.e., MDH, P12, HYB and LGT; described below).

We could not detect recombined sequences computationally in MDH, ATC and LGT by any of the methods used. P12 and HYB showed recombination by only a single method and therefore the evidence was not considered compelling [59]. Multiple methods detected three breakpoints caused by two recombination events within CTV11 sequence, from aligned positions 296–437 (first event: Bootscan p = 1.5×10^−3^ and 3seq p = 2.9×10^−6^) and from positions 455–987 (second event: MaxChi p = 8.6×10^−3^ and Chimaera p = 4.4×10^−3^). Hence, the full sequence of CTV11 was not used in the coalescence simulation study; only a non-recombining portion was included for data analysis (see Methods).

The population structure analysis of SSR marker data, carried out using the admixture model and correlated allele frequency option, found that K = 6 showed the highest posterior probability; thus, estimations of subpopulation memberships and ancestry were calculated assuming K = 6 (for details, see Figure S6). Most analyzed accessions (included in the current study) found to be pure mandarins by STRUCTURE analysis (Figure S6) were clustered in the same supported clade in the HYB (PP 1.00; Figure 1), LGT (PP 0.99; Figure 2) and MDH (PP 1.00; Figure 3) gene trees. South Coast Field Station was the only pure citron (as determined by STRUCTURE) that was included in the sequence analysis (Figure S6). At least one allele from Arizona citron and Frost Eureka lemon accessions was always found in a citron clade in each of the four informative genes (PP 1.00 in each: Figures 1, 2, and 3, Figure S5). At least one allele from Palestine sweet lime also clustered with core citrons in four of the trees. Three pummelos (Kao Pan, Kao Panne and Mato Buntan), each apparently pure according to the STRUCTURE results, contributed at least one allele to a common pummelo clade in each informative gene (PP 0.98–1.00; Figures 1, 2, and 3, Figure S5). Only two trifoliate orange (*Poncirus trifoliata*) accessions were sampled, and they form a well supported clade in all six genes (PP 1.00; Figures 1, 2, and 3, Figures S2, S3, S4, and S5). Despite the overall poor bootstrap values in ATC tree (Figure S3), all trifoliate orange alleles form a well supported clade.

In contrast to the core clades of mandarin, citron, pummelo and trifoliate orange, relationships among accessions classified as kumquat and papeda were less clear. The only kumquat sampled here (Nagami) was not always separate from the core clades. Some alleles of Nagami kumquat, Winged lime, Nasnaran mandarin, Kalpi lime and Mountain citron accessions were often found together in each gene. Some alleles of these accessions were usually found with core mandarins (Figures 1, 2, and 3, Figures S2 and S5).

### Phylogenetic Indications of Hybrid Origin

Several accessions derive their alleles from only two core clades, sometimes as heterozygotes, and thus are likely to be of hybrid origin: Palestine sweet lime and Frost Eureka lemon (both citron-mandarin), Bouquet de Fleurs sour orange and Rubidoux sour orange (both mandarin-pummelo). Other accessions consistently derive their alleles from one core clade and another unknown clade, often with heterozygosity, also indicating a hybrid origin: Indian sour citron and Mexican lime (both citron-and other), Winged lime, Nasnaran and Korai mandarin (all mandarin-and other). In some cases, these ancestries are not consistent with the results of STRUCTURE analysis; e.g., STRUCTURE shows Rubidoux sour orange as a mandarin-papeda mix and King mandarin appears to be over 70% papeda without any indication of this in the gene trees sampled here.

### Paralogy in CTV11

The DNA distance between sweet orange and trifoliate orange ranged from 0.015–0.017 in MDH, 0.014–0.022 in ATC, 0.015–0.020 in P12, 0.019–0.020 in LGT, 0.029–0.032 in HYB, to 0.057 in CTV11. Although this comparison is between accessions that span the root node in the first five genes, they do not do so in the last. The distance between pummelo and trifoliate orange does span the root node in CTV11 (Figure S2) and ranges from 0.116–0.121.

The CTV11 topology may be the result of paralogy caused by gene duplication and loss. Even after analysis without the recombined portions, trifoliate oranges are nested shallowly within the tree rather than being a sister clade to the rest of the *Citrus* group (Figure S4). This suggests that some of the relationships found in CTV11 are not reliable. Further evidence from the rate of change supports the hypothesis that CTV11 might be affected by mistaken orthology. The genetic distance between sweet orange and trifoliate orange in each gene suggests that these genes are evolving with somewhat similar rates, although CTV11 is the fastest (2–4 times faster). When rates involving the root node of *Citrus* s.l. are compared (by using trifoliate – pummelo distances in CTV11), CTV11 appears to be evolving between 4 times to 7.5 times faster than the other genes. The second fastest gene, HYB, is only changing twice as fast as the slowest genes; thus it seems that the substitution rate of CTV11 is unusual.

### Methods of Hybrid Detection

Median *N_e_* (effective population size of gene copies) values were derived from θ and mutation rate and ranged from c. 2,000 to 21,000 (Table 3) for the first species tree listed in the in the following sections, with a cross-loci mean of medians between c. 4,000 and 13,000. Importantly, ancestral *N_e_* (c. 4,000–5,000) was lower than extant species’ *N_e_* (c. 5,000–13,000), the latter indicative of post-speciation population expansions that do not affect the probability of lineage sorting among these species. For our analysis, we chose to use a value that is conservative with respect to ancestral *N_e_* (i.e., 8,000). Alternative species trees used in MCMCcoal (see Appendix S1) did not substantially affect *N_e_* (not shown), thus the estimate of *N_e_* appears to be robust to error in the assumed species tree.

**Table 3:** Data used in the estimation of effective population size (*N_e_*).

Statistic	HYB	LGT	MDH	Mean *N_e_*
mean p-distance^a^	0.0336	0.05	0.0227	
μ^b^	3.31*10^−9^	4.24*10^−9^	1.93*10^−9^	
corrected μ^c^	6.62*10^−8^	8.47*10^−8^	3.85*10^−8^	
θ^d^ Citron	0.00314 (n = 6)	0.00133 (n = 8)	0.00311 (n = 7)	
θ Mandarin	0.00240 (n = 18)	0.00317 (n = 17)	0.00330 (n = 22)	
θ Pummelo	0.00287 (n = 8)	0.00203 (n = 7)	0.00102 (n = 5)	
θ Trifoliate	0.00192 (n = 5)	0.00069 (n = 2)	0.00076 (n = 4)	
θ MRCA^e^ all	0.00122	0.0011	0.00092	
θ MRCA Pummelo, Citron, Mandarin	0.0014	0.00111	0.00076	
θ MRCA Citron, Mandarin	0.00108	0.00069	0.00095	
*N_e_* f Citron	11,867 (6,767–20,799)	3,938 (1,705–9,077)	20,210 (11,137–34,773)	12,005
*N_e_* Mandarin	9,068 (4,643–15,672)	9,360 (5,222–15,721)	21,384 (12,182–35,377)	13,271
*N_e_* Pummelo	10,855 (5,728–18,778)	5,980 (2,537–12,015)	6,587 (1,642–16,244)	7,807
*N_e_* Trifoliate	7,262 (3,506–14,569)	2,024 (469–6,384)	4,913 (1,116–14,927)	4,733
*N_e_* MRCA all	4,609 (824–13,307)	3,236 (628–8,626)	5,945 (610–17,841)	4,597
*N_e_* MRCA Pummelo, Citron, Mandarin	5,271 (1,243–15,147)	3,269 (628–8,301)	4,913 (201–16,556)	4,484
*N_e_* MRCA Citron, Mandarin	4,077 (707–12,298)	2,047 (316–7,230)	6,146 (980–17,069)	4,090
Overall mean *N_e_*				7,284

a Changes per site of non-coding sequences only between *Poncirus trifoliata* cultivars ‘Pomeroy’, ‘Flying Dragon’ and *Citrus sinensis* cultivar ‘Washington’ navel orange calculated using MEGA with complete deletion of indels (indels were included as separately coded single characters).

b Neutral mutation rate (changes per site per year) on the assumption that non-coding sequences are neutrally evolving and that *P. trifoliata* and *C. sinensis* diverged 5.9 Ma.

c μ/20 converts the per year value into a per generation value, assuming a generation time of 20 years.

d Median estimated values using MCMCcoal with allele samples size indicated for each gene.

e MRCA = most recent common ancestor.

f **Median** (Lower – Upper) 95^th^ percentile range.

**Figure 4:**
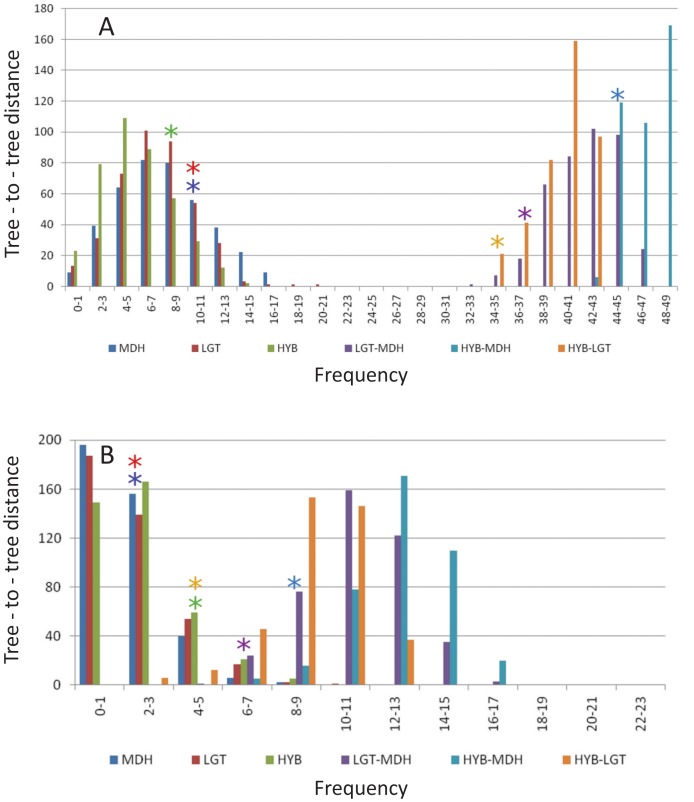
Null and observed genes tree-to-tree distance distributions. (A) Tree-to-tree distances between 20 trees drawn from the posterior distribution of each observed gene tree from one another (right hand side; 400 combinations per pair wise comparison) and the observed gene trees to 20 simulated gene trees that makes up the null distribution for each observed gene tree (left hand side). The position of the 80% critical value for each of the three null distributions is marked (blue: MDH, red: LGT and green: HYB asterisks; left hand side). The lower boundary of the 95% credibility interval of each observed pair of genes’ tree-to-tree distances are marked (purple: LGT-HYB, light blur: HYB-MDH and orange: HYB-LGT asterisks; right hand side). The test statistic, φ, is the distance between the closest left and right hand asterisks and does not overlap, thereby indicating a rejection of the null hypothesis of lineage sorting alone. (B) Tree-to-tree distances as in A, but after the removal of individuals with a putative hybrid origin. Asterisks also as in A. In this case, removal of individuals of putative hybrid origin results in a zero result for φ, thereby indicating that the null hypothesis of lineage sorting alone cannot be rejected. The interpretation is that those individuals that have been removed (compared to A) require an alternative explanation beyond lineage sorting in order to account for their incongruent placement among the three gene trees, whereas those that remain in B do not.

New “gene” trees were simulated in Mesquite using 8,000 individuals (handled as haploids, thus individuals = gene copies). When distances among gene trees and their null distributions were plotted, we found that φ >0 in each comparison, thereby rejecting the null hypothesis of lineage sorting alone as the explanation of gene tree incongruence (Figure 4A).

We discovered that 14 individuals needed to be removed in order to have φ ≤0, namely Bouquet de Fleurs sour orange, Frost Eureka lemon, Mountain citron, Ichang papeda, Indian sour citron, Mexican lime, Nagami kumquat, Rubidoux sour orange, Winged lime, Nasnaran mandarin, Kalpi lime, Palestine sweet lime, Korai mandarin and Finger lime (Table S4, Figure 4B). These particular individuals cause the test to reject the null hypothesis. Removal of a randomly chosen but equal number of individuals to the putative hybrid set still resulted in φ >0 (Table S4), indicating that hybrids were still present in these 10 randomly chosen taxon sets. This shows that the test discriminates among individuals and can indicate which individuals are most likely to be of hybrid origin.

### Species Tree Inference

Both the three locus and four locus analyses using “Deep Coalescence Multiple Loci” to infer species relationships returned the following single tree: (trifoliate, (pummelo, (citron, mandarin))) = species tree A. The five locus analysis also returned this tree as one of three alternatives. The six locus analysis (with the paralogous CTV11) returned a different single tree: (pum., (cit., (trifol., man.))) = species tree B. Tree B was not one of the alternatives in the five locus analysis.

The DNA distance between sweet orange and trifoliate orange ranged from 0.015–0.017 in MDH, 0.014–0.022 in ATC, 0.015–0.020 in P12, 0.019–0.020 in LGT, 0.029–0.032 in HYB, to 0.057 in CTV11. Although this comparison is between accessions that span the root node in the first five genes, they do not do so in the last. The distance between pummelo and trifoliate orange does span the root node in CTV11 (Figure S2) and ranges from 0.116–0.121.

### Linkage among the Six Nuclear Loci

MDH and P12 mapped to scaffold 1 of the haploid Clementine mandarin genome at approximately 26% and 96% of the chromosome length indicating no linkage. CTV11 and LGT mapped to scaffolds 7 and 8 respectively. HYB and ATC mapped to scaffold 9 at 94% and 97% of the chromosome length indicating close linkage. Since ATC was not included in most of the analysis due to high homoplasy index, the five genes used can be considered to be “unlinked”. All six genes had only one blast hit indicating that they are single copy genes, although portions of CTV11 had good (>80% identity) matches to additional genes.

## Discussion

### Methods of Hybrid Detection

Gene genealogies can often differ from species phylogenies. Dissimilarity in the topology of phylogenetic trees constructed from different genes has been reported from many systems [60]. A similar phenomenon is observed in citrus. Two major causes of such incongruence are: hybridization and lineage sorting. Identification of the cause for incongruence can facilitate better understanding of citrus phylogeny.

Maureira-Butler et al. [38] describe a method that distinguishes between hybridization and lineage sorting using incongruent gene tree data from two nuclear genes. In this study, we extend Maureira-Butler’s test to a dataset comprised of three gene trees, with a preliminary evaluation of the type 1 error rate associated with a given critical value used in the test. The critical value decreased from 100% to 80%, representing a substantial improvement in the sensitivity of the test.

Despite the increased sensitivity of the test, it is likely that it remains conservative when a third gene is added. Any pair of genes’ observed distances overlapping the null distribution will cause the test to no longer reject the null hypothesis. This was initially done to accommodate data from genes which may not be representative samples of the species trees. However, if none of the genes used are outliers, then when any two genes no longer show differing topological positions for a hybrid (but still differ in the third tree), the test will not reject the null hypothesis. Hence this represents a very conservative case favoring retention of the null hypothesis. Our preliminary simulations found that an 80% critical value gave <5% type 1 error rate. Most individuals suspected of having hybrid origins (13 out of 14) would still have been inferred as such using a critical value of >98.5%. For these reasons we believe that the hybrid detection is probably robust for most, if not all, cases discussed here. This conclusion is supported by the congruence among phylogenies with patterns expected under hybridization (repeated placement in two alternative positions among a collection of gene trees), and by the congruence with STRUCTURE results, especially for individuals with recent hybrid histories.

Although there are now several methods available using trees for distinguishing between hybridization and incomplete lineage sorting [61], [62], our method differs in two ways from other approaches. Firstly, approaches that explicitly model hybridization are expected to suffer from a loss of power with the addition of more hybridization events in a dataset. This is because each hybridization event is described with additional parameters unique to each event [63]. The approach we used here does not model hybridization *per se*. The difference between the observed gene trees and null distributions is expected to simply increase as additional hybridization events are present. This is because these additional events have a larger effect on the topological incongruence compared with incomplete lineage sorting. Thus, there may be an increase in power to detect hybridization in a collection of gene trees.

The second difference is that, unlike other approaches, the null expectation here is derived from the observed gene trees and not from a species tree [62], [64]. Gene sequence data used to infer a species tree may be affected by hybridization, but this is not known *a priori*. Until this has been established, an inferred species tree must be treated with suspicion. The inclusion of sequences produced under simulated hybridization events can produce a misleading (but well supported) inference of the species tree (unpublished results). If the species tree is incorrect, simulating coalescent null distributions from this inferred (and incorrect) species tree will not provide correct assessment of the occurrence of hybridization. Our approach seeks to avoid this circularity, although there may be other biases introduced by directly using gene trees to produce null distributions. Thus we expect that the approach used here has favorable properties for diagnosing gene trees that differ because of hybridization.

### Analysis based on STRUCTURE, Phylogenetic Trees and Coalescence Studies

We reanalyzed published SSR data [24] with STRUCTURE 2.3 but edited the dataset to contain fewer individuals known to have admixtures. We reasoned that if a dataset included a larger proportion of admixed individuals relative to those that do not have admixture, it may be difficult to accurately determine the number of ancestral groups because the dataset as a whole will be similar to a single panmictic population. Although the previous analysis by Barkley [24] was consistent with many expectations, a few notable discrepancies motivated our reanalysis. For example, pummelo was expected to be a contributor to the sweet orange genome but was not revealed as such in the previous study [24].

In our analysis, the K value was determined to be six (citron, mandarin, pummelo, papeda, kumquat and trifoliate orange). Previous analysis revealed only five of these groups since kumquats could not be distinguished from papedas [24]. The result of the current STRUCTURE analysis is, in our opinion, more representative of the populations. Although most of our phylogenetic inference rested on the gene tree results, STRUCTURE analyses supported and complemented several of our conclusions. Of the putative hybrids, eight appear to be recent admixtures containing a mixture of alleles from two main sources as identified by STRUCTURE (Figure S6, Table S6; Mountain citron, Indian sour citron, Rubidoux sour orange, Palestine sweet lime, Nasnaran mandarin, Bouquet de Fleurs sour orange, Frost Eureka lemon and Mexican lime. Two cultivars showed admixture, but with most alleles derived from a single source based on the STRUCTURE results (Winged lime and Korai mandarin), confirming the analyses of gene trees. Three putative hybrids (Kalpi lime, Nagami kumquat, Ichang papeda), however, do not show signs of admixture in STRUCTURE, containing >98% alleles from a single source. In the former two groups of putative hybrids we can confidently rule out mistaken orthology as a cause of incongruence, because the STRUCTURE results also support our conclusion of hybridization. In the latter group (Kalpi lime, Nagami kumquat and Ichang papeda), the hybrid history predicted by coalescence analysis is supported by the repeated hybrid-type of phylogenetic pattern and high heterozygosity. For these three cultivars, we can confidently rule out other causes of incongruence.

Ichang papeda and Nagami kumquat show a complex pattern that cannot be explained by lineage sorting alone. Both have alleles in the shallow mandarin clade in one gene each (which is consistent with recent hybridization and difficult to reconcile with lineage sorting), but there is no consistent second parental lineage. A more complex hybridization scenario (involving more than two lineages) might explain these results. Our results are consistent with the assumptions of Abkenar et al. [16] who considered *C. ichangensis* to be a hybrid of a mandarin and a papeda.

Nagami kumquat alleles group in shallow positions with mandarins and also with Mountain citron, but other placements are rather deep. Even the well supported relationship between Nagami kumquat alleles and citron alleles in CTV11 has a deep divergence between them. It is not clear what the causes of the incongruence among gene trees are for this accession, although some admixture with mandarin seems likely and might be driving the coalescent test result.

The CTV11 topology, however, may be the result of paralogy or gene duplication. Even after analysis without the recombined portions, trifoliate oranges are nested shallowly within the tree rather than being a sister clade to the rest of the *Citrus* group. The other genes place trifoliate orange sister to the rest of *Citrus* group (recognized by Bayer et al. as *Citrus* s.l.) or in a position that cannot exclude this placement (i.e., due to lack of support). This position is also found in the BA of the most comprehensive cpDNA analysis to date, if branches with less than 0.95 PP are collapsed [10]. This suggests that some of the relationships found in CTV11 are not reliable. CTV11 is an NBS-LRR disease resistance gene, a class of genes known to exist as a large gene family in plants and suspected to undergo rapid duplication and loss of copies [65], [66]. Our blast analysis with the complete genome of haploid Clementine mandarin indicated presence of only one copy of this sequence. However, the copy number in other accessions could differ as could the historical copy number in a common ancestor.

### Phylogeny of Citrus

We present a hypothesis of phylogenetic relationships among species of *Citrus* that accounts for both hybridization and lineage sorting. In the present study a limited number of species have been sampled and, after excluding individuals of hybrid origin, we are left with a small number of individuals per species. We believe that the methods described here can be the foundation for a more robust estimate of species phylogeny in future studies of *Citrus.*

Species tree A (trifoliate,(pummelo,(citron, mandarin))) appears to be the best explanation of the fit of gene trees to a species tree while minimizing deep coalescences, as it was found in the most informative loci. We also found this tree in the five locus analysis as one of three alternative solutions. The inclusion of possibly paralogous CTV11 changed the species tree estimation to reflect that gene, placing trifoliate orange (*Poncirus*) sister to mandarin. This latter tree was not found among the alternative trees in the five locus analysis. Analysis of a few individuals reduces the chance of detecting alternative positions that alleles might occupy and thereby reduces the accuracy of the deep coalescence approaches. Despite this, simulations [57] suggest that we have favorable conditions for finding the species tree: a moderate tree depth (c. 350 K generations), an average of c. 3 individuals per species and information from six loci.

A lingering concern is that the species tree may be driven by the more resolved gene HYB tree alone. Thus, *Citrus* phylogeny can be improved by using loci that generate more resolved gene trees, greater sampling of individuals within species and inclusion of more species. Further analyses using the coalescent to reconstruct the species tree are also desirable, but may only be worthwhile when these issues have been resolved.

### Origins of Specific Citrus Cultivars

A general issue that limits the power of PCR-based sequence analysis is that primers may not amplify some alleles in certain taxa and therefore the amount of hybridization may be underestimated. Similarly, in clonally propagated cultivars, particularly those selected for low seed content, chromosomal rearrangements may occur that create deletions, making the cultivar hemizygous. The extent to which these factors affect this study is unknown, although RFLP analysis of the loci sequenced might reveal heterozygosity not detected in the PCR products sequenced. These and other complexities suggest that patterns of heterozygosity observed for a hybrid may not always perfectly match those expected based on its putative ancestry.

Barrett and Rhodes [2] suggested that the sweet oranges are predominantly a mandarin genotype introgressed with pummelo genes. Later work using RAPDs, SCARs and cpDNA fragment analysis indicated that sweet oranges derived 50% of nuclear markers from pummelo and 50% from mandarins, and that pummelo is probably the female ancestor [23]. The female ancestry of sweet orange among pummelos is supported by cpDNA sequences [10], whereas all six nDNA genes here show that sweet orange (Washington navel) alleles do not cluster with the pummelo group, instead they are placed with mandarins or not clearly resolved. King mandarin was thought to be a natural hybrid between *C. reticulata* (mandarin) and *C. sinensis* (sweet orange) [1], a hypothesis consistent with our results. The STRUCTURE analysis shows pummelo and mandarin admixture in Washington Navel orange [21] (sample no. 76 in Figure S6 is Tarocco blood orange, considered to be very similar to Washington Navel orange). However, a sample of six genes, as studied here, may be inadequate to detect pummelo alleles if sweet orange was formed by the backcrossing of a pummelo X mandarin hybrid to mandarin [70] or a more complex series of intercrosses.

Lemons are thought to be hybrid accessions with limited genetic diversity with citron contributing a major portion of the genome as the male parent [18], [67]. Swingle treated it as a distinct species close to citron but he also acknowledged a strong possibility that it could be of hybrid origin [1]. Analysis of cpDNA indicated that the female parent of the Eureka lemon might be a pummelo or a pummelo-like citrus [10], [15]. Citron, pummelo and mandarin ancestry for lemons was implied in a study of ISSRs [67]. The only lemon used here, Frost Eureka, is of hybrid origin (see above). However, it does not appear to be a simple F1hybrid, as it is homozygous at two of six loci.

Swingle treated the lime (*C. aurantifolia*) as a distinct species and thought that it originated in the East Indian Archipelago and was moved to the Asian mainland and beyond by humans [1]. However, Barkley et al. [24] observed that 27 lime accessions appeared heterozygous and there was evidence of admixture from pummelo and/or mandarin, although citron was the dominant source of alleles. Among the four limes analyzed here (Kalpi lime, Winged lime, Palestine sweet lime and Mexican lime), we confirm a hybrid history for all of them, except for Kalpi lime which was mostly made up of Papeda alleles based on the STRUCTURE analysis. We did not find pummelo alleles in Palestine sweet lime as reported earlier [18], [24], instead we found only citron and mandarin alleles, including heterozygosity in two of the genes (Table S2).

The sour oranges were considered by Swingle to be a distinct species, *C. aurantium* [1]. However, both sour oranges we studied (Bouquet des Fleurs and Rubidoux) were found to be of hybrid origin, with pummelo and mandarin parentage. The two sour orange accessions appeared to be admixed in STRUCTURE analysis (Figure S6), with the pummelo as the contributor of maternal alleles based on cpDNA results [10].

The Swingle system recognizes two subgenera in the genus *Citrus* s.s. Subgenus *Papeda* has six species and subgenus *Citrus* has 10. This classification is based on the occurrence of a broad-winged leaf petiole and acrid oil in the fruit of the former subgenus. Subgenus *Papeda* includes *C. ichangensis*, *C. latipes*, *C. micrantha*, *C. celebica*, *C. macroptera* and *C. hystrix*. Of the papedas and probable papedas used here, we found that Ichang papeda (*C. ichangensis*) and Hanayu (*C. hanaju*) might be of hybrid origin, containing mandarin alleles in addition to papeda alleles – as also observed in cpDNA analyses [18]. Hanayu appears to be a hybrid consisting of papeda, mandarin and some other citrus alleles (Table S2, Figures 2–3 and Figures S3, S4, and S5). Since data from one of the most informative loci, Hyb, was not available for Hanayu papeda, we could not include it in the coalescence test. As in kumquats, the alleles do not fit into core clades suggesting that these accessions are carrying some alleles that are unique to them. This is consistent with the idea that papeda is a separate species and that mandarin has contributed to the parentage of both these accessions, even though it is not evident in the STRUCTURE results.

Mountain citron (*C. halimii*) was considered to be one of the allopatric species of *Citrus* from the tropics [3], [68], [69]. RAPD and RFLP analyses suggest that *C. halimii* may be very similar to some of the papeda species [21]. However, in our analyses, Mountain citron alleles clustered with either Nagami kumquat, mandarin, or with other groups. The hybrid origin of this accession (and potentially the whole species) is supported by our coalescence test. Scora [68] noted that “the high amounts of+limonene (±90%) in the rind oil of *C. halimii* sets it apart from … the acid member group, and places it closer to the mandarin-sweet orange assemblage of which such high amounts of+limonene are characteristic”. This observation is in accord with mandarin alleles found in *C. halimii* in one gene in the present analysis. Scora [68] also noted that some isozyme alleles found in *C. halimii* were shared with kumquat, again consistent with our result and supporting the possible hybrid origin of *C. halimii*. Since *C. halimii* alleles were not consistently placed in any core clade, it may indicate that a distinct species or group of species exists as a potential allele donor. Our sample size is small, limiting the power to detect such a group. A kumquat origin is supported tentatively here and by the STRUCTURE results (Table S6).

### Conclusions

We present a preliminary phylogeny of some species of *Citrus* that takes into account many factors that may confound phylogenetic inference. Improved prospects for the estimation of the species phylogeny in *Citrus* clearly require sampling more species, especially Australasian and New Caledonian ones, as well as more individuals of the species sampled here. However, the coalescence analysis conducted here combined with the phylogenetic tree data and STRUCTURE results may be useful to untangle many phylogenetic questions pertaining to *Citrus*.

## Supporting Information

Figure S1 **A**: Majority rule consensus tree of 20 trees drawn from the stable Bayesian posterior distribution of the HYB analysis that were used as individual input trees to the coalescence test (via smoothing in r8s, etc). Individuals inferred to have a hybrid origin are marked with an asterisk. **B**: Majority rule consensus tree of 20 trees drawn from the stable Bayesian posterior distribution of the LGT analysis that were used as individual input trees to the coalescence test (via smoothing in r8s, etc). Individuals inferred to have a hybrid origin are marked with an asterisk. **C**: Majority rule consensus tree of 20 trees drawn from the stable Bayesian posterior distribution of the MDH analysis that were used as individual input trees to the coalescence test (via smoothing in r8s, etc). Individuals inferred to have a hybrid origin are marked with an asterisk.
(PDF)

Figure S2 **Bayesian consensus phylogram of CTV11 sequences from** ***Citrus*** **and related genera.** Only the first 295 nucleotides used to avoid recombined parts of this locus. Clades with posterior probabilities (PP) of 0.95–1.00 are marked with bold branches; clades with PP less than 0.95 but at least 0.90 are shown above or to the left of branches. Accessions belonging to *Citrus, Poncirus, Fortunella* and *Microcitrus* are indicated by cultivar or common names as indicated in Table 1. Latin names are used for outgroup genera only. Suffix A and B refer to the two haplotypes. Accessions without a suffix have only one haplotype. The traditional cultivar group to which the accession was previously assigned is indicated by three letter abbreviation following the cultivar/haplotype information. The abbreviations used are: CIT: citron; MAN: mandarin; PUM: pummelo; TRF: trifoliate orange; SWO: sweet orange; KUM: kumquat; SOR: sour orange; PPD: papeda; LIM: lime and lemon. Cultivars that indicated an admixture of more than 10% in STRUCTURE analysis (Table S6) are marked by an asterisk. Scale bar for branch lengths represents substitutions per site.
(PDF)

Figure S3 **Bayesian consensus phylogram of ATC sequences from** ***Citrus*** **and related genera.** Clades with posterior probabilities (PP) of 0.95–1.00 are marked with bold branches; clades with PP less than 0.95 but at least 0.90 are shown above or to the left of branches. Other details as per Figure S2. Groups of alleles discussed in the text are marked by cultivar grouping names.
(PDF)

Figure S4 **Bayesian consensus phylogram of CTV11 sequences (using all nucleotides) from** ***Citrus*** **and related genera.** Clades with posterior probabilities (PP) of 0.95–1.00 are marked with bold branches; clades with PP less than 0.95 but at least 0.90 are shown above or to the left of branches. Other details as per Figure S2. Groups of alleles discussed in the text are marked by cultivar grouping names.
(PDF)

Figure S5 **Bayesian consensus phylogram of P12 sequences from** ***Citrus*** **and related genera.** Clades with posterior probabilities (PP) of 0.95–1.00 are marked with bold branches; clades with PP less than 0.95 but at least 0.90 are shown above or to the left of branches. Other details as per Figure S2. Groups of alleles discussed in the text are marked by cultivar grouping names.
(PDF)

Figure S6 **Analysis of 212 Citrus accessions to infer populations by STRUCTURE analysis.** Cultivar information is in Table S5. Numbers 2–10 represent Kumquats; 11–36 = limes, lemons and citrons; 37–116 = mandarins; 117–139 = papedas; 140–203 and no. 1 = pummelos; 204–212 = trifoliates. The numbers in parenthesis indicate assumed population groups. Y axis =  probable admixture in each accession. Green =  kumquat; blue = citron; pink = mandarin; dark blue = papeda; red = pummelo; yellow = trifoliate. One million iterations were run after 500,000 iterations were discarded as “burnin”. Populations assumed, K = 6. Three letter abbreviations are indicated for taxa included in the SNP analysis. Samples 76, 93, 206 and 208 are, Tarocco (TRO), Neopolitana (NPL), Fairhope (FHP) and English Dwarf (EDW) considered equivalent to Washington navel orange (WNO), Frost Owari Satsuma (FOS), Flying Dragon (FDR) and Pomeroy (PMY) respectively.
(PDF)

Table S1 **Harmonic means of log likelihoods for each model used for each gene for Bayesian analyses based on nruns = 2 and nchains = 10.** Model details and the individual trees for each analysis are in Figs 1, 2, and 3 and figures S2, S3, S4. and S5. SD = average standard deviation of split frequencies. Bold likelihoods indicate the model preferred by Bayes factors.
(PDF)

Table S2 **Inferred allele compositions using Bayesian and parsimony data.** Alleles were designated with uppercase letters (in recognized clades with PP>95) or lowercase letters (in clades with PP 80–95). All others were considered to be of unknown origin. Abbreviations used: C = citron; M = mandarin; P = pummelo; D = papeda; K = kumquat; A = Australasian; T = Trifoliate and U = unknown. The four outgroup accessions, *Atalantia ceylanica*, *Swinglea glutinosa*, *Hesperethusa crenulata* and *Citropsis gabunensis* consisted of unknown alleles. ATC sequences had high homoplasy; allele assignments are less probable than for other genes.
(PDF)

Table S3 **Parsimony-based tree characteristics for the six citrus genes studied.**
(PDF)

Table S4 **Difference in tree to tree distance scores between 95% CI of observed gene tree distances from one another and the simulated “gene” tree distances from observed gene trees, under various taxon exclusion sets.** *14 putative hybrids (or linegaes of hybrid origin) were removed and compared with 14 randomly removed taxon sets. A *p* value of <0.05 rejects the null of lineage sorting alone as an explanation for incongruence among observed gene trees. In these cases hybrids remain in the taxon set. The 14 putative hybrids appear to be the smallest set of taxa that can be removed and fail to reject the null, so therefore are the best candidates for having a hybrid origin. Individuals are: 1 ARZ,2 IND,3 SCF,4 HLM,5 KLP,6 WGL,7 MEX,8 PAL,9 FRS,10 AMB,11 TNC,12 SEM,13 ENC,14 NIP,15 CLP,16 BDF,17 RBD,18 WNO,19 KNG,20 KPN,21 KPE,22 MBM,23 ICH,24 FDR,25 PMY,26 NGM,27 MIC.
(PDF)

Table S5 **Accessions included in the STRUCTURE analysis.** For the SSR analysis, we included a subset of 212 accessions from a total of 370 accessions reported by Barkley et al (2006). Many hybrids were excluded from the analysis. Accessions with an asterisk (*) were also included in the SNP analysis. Accessions with two asterisks (**) were selected as being very similar to Parent Washington navel orange (CRC 3596, Tarocco), Frost Owari Satsuma (CRC 3848, Neopolitana), Flying Dragon (CRC 3351, Fairhope) and Pomeroy trifoliate (CRC 3876, English Dwarf).
(PDF)

Table S6 **Inferred ancestry of individuals included in Structure and SNP analysis.** Numbers in columns 5–11 represent proportion of alleles scored based on STRUCTURE and SNP values (in parenthesis). ^a^ Represents the identifying numbers used in Structure analysis (Fig S6). ^b^ Citrus variety collection reference numbers. ^c^ Tentative groups recognized by Barkley et al [21]. Other abbreviations used: PUM = pummelo; KUM = kumquat; PPD = papeda; TRF = trifoliate; MAN = mandarin; CIT = citron. Column designated “other” refers to alleles that were not assigned to any of the other recognized groups in SNP analysis. *Accessions with more than 10% admixture by STRUCTURE. # Hybrids according to coalescence analysis but not by STRUCTURE analysis.
(PDF)

Appendix S1 **Methods used for coalescent simulation analyses are described in detail.**
(DOC)
