# Supplementary figures and images for: A Six Nuclear Gene Phylogeny of Citrus (Rutaceae) Taking into Account Hybridization and Lineage Sorting
Source: PLoS One. 2013 Jul 16;8(7):e68410. doi: 10.1371/journal.pone.0068410 (PMC3713030; doi:10.1371/journal.pone.0068410)

Figure S1A

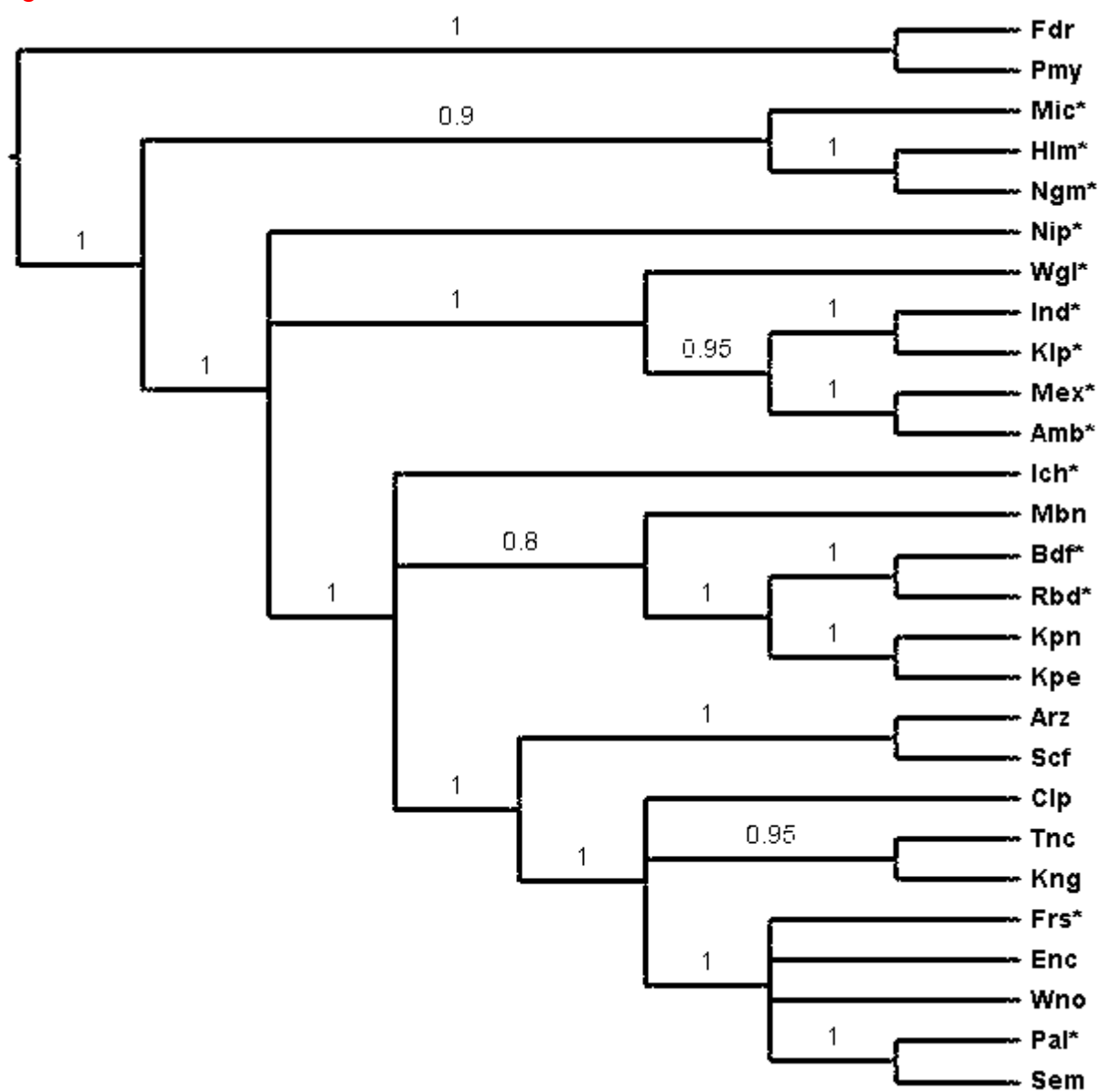

Figure S1B

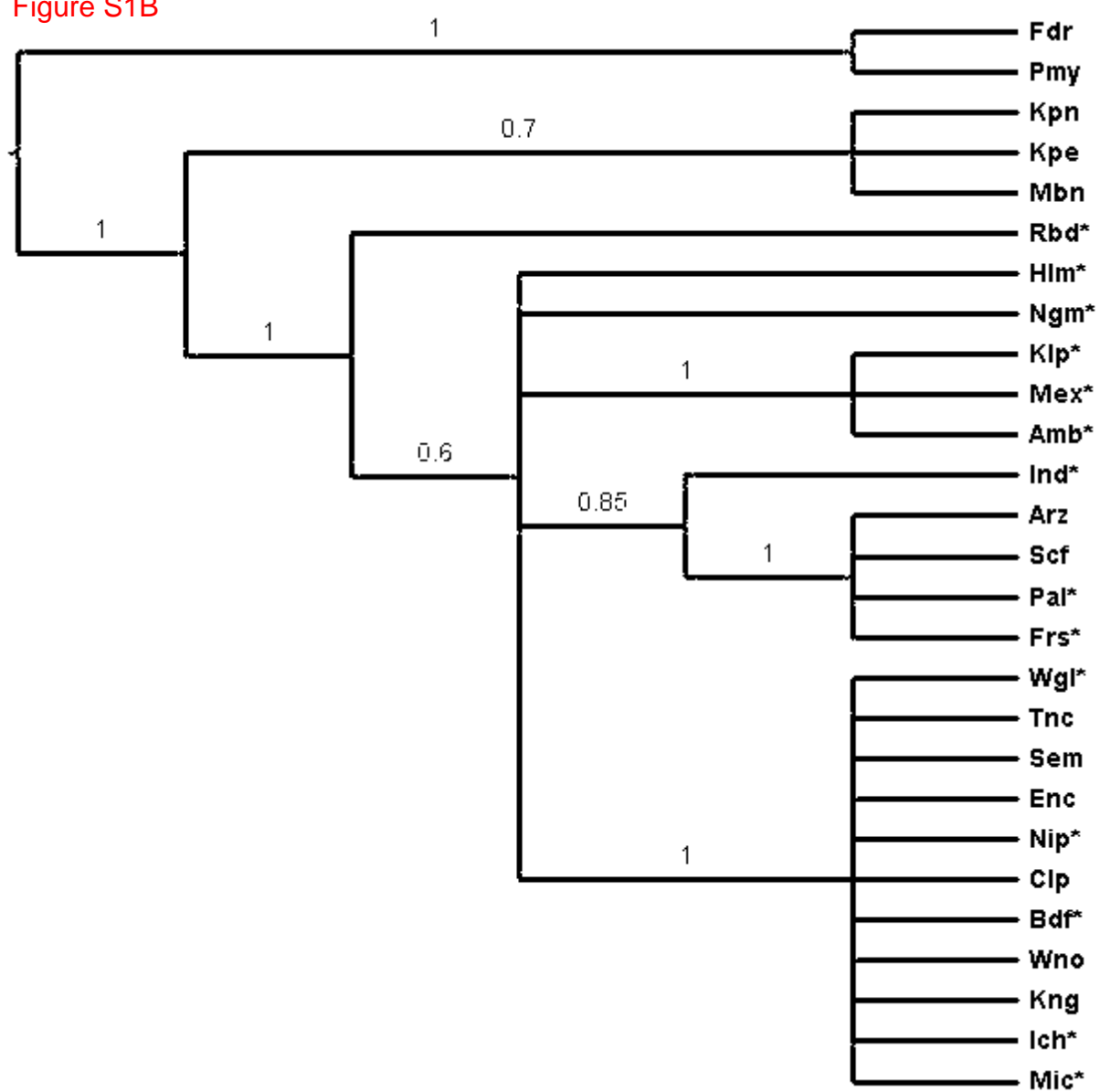

Figure S1C

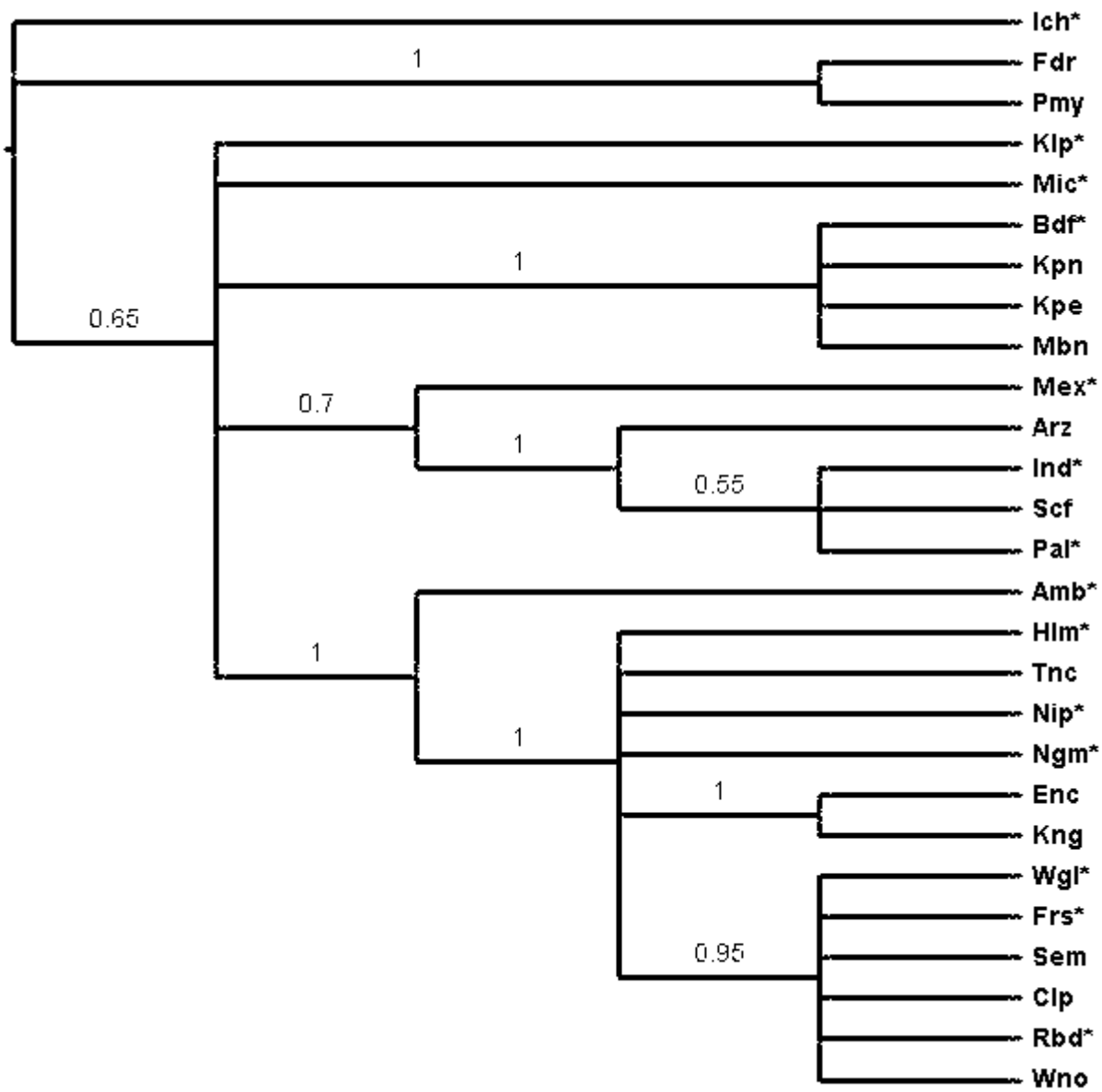

Supplement: Figure S1 — A: Majority rule consensus tree of 20 trees drawn from the stable Bayesian posterior distribution of the HYB analysis that were used as individual input trees to the coalescence test (via smoothing in r8s, etc). Individuals inferred to have a hybrid origin are marked with an asterisk. B: Majority rule consensus tree of 20 trees drawn from the stable Bayesian posterior distribution of the LGT analysis that were used as individual input trees to the coalescence test (via smoothing in r8s, etc). Individuals inferred to have a hybrid origin are marked with an asterisk. C: Majority rule consensus tree of 20 trees drawn from the stable Bayesian posterior distribution of the MDH analysis that were used as individual input trees to the coalescence test (via smoothing in r8s, etc). Individuals inferred to have a hybrid origin are marked with an asterisk. (PDF) [file pone.0068410.s001.pdf]

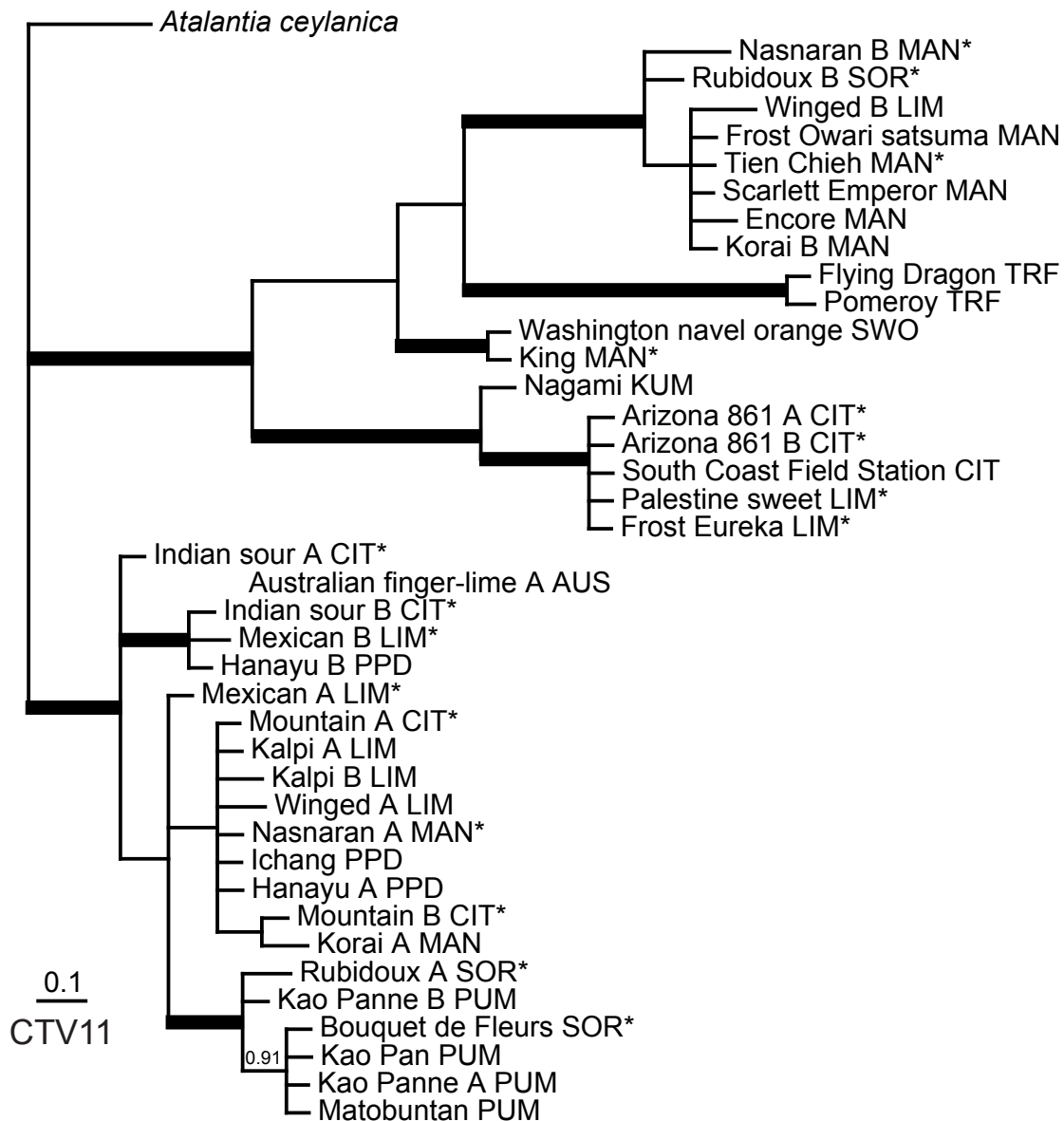

Supplement: Figure S2 — Bayesian consensus phylogram of CTV11 sequences from Citrus and related genera. Only the first 295 nucleotides used to avoid recombined parts of this locus. Clades with posterior probabilities (PP) of 0.95–1.00 are marked with bold branches; clades with PP less than 0.95 but at least 0.90 are shown above or to the left of branches. Accessions belonging to Citrus, Poncirus, Fortunella and Microcitrus are indicated by cultivar or common names as indicated in Table 1. Latin names are used for outgroup genera only. Suffix A and B refer to the two haplotypes. Accessions without a suffix have only one haplotype. The traditional cultivar group to which the accession was previously assigned is indicated by three letter abbreviation following the cultivar/haplotype information. The abbreviations used are: CIT: citron; MAN: mandarin; PUM: pummelo; TRF: trifoliate orange; SWO: sweet orange; KUM: kumquat; SOR: sour orange; PPD: papeda; LIM: lime and lemon. Cultivars that indicated an admixture of more than 10% in STRUCTURE analysis (Table S6) are marked by an asterisk. Scale bar for branch lengths represents substitutions per site. (PDF) [file pone.0068410.s002.pdf]

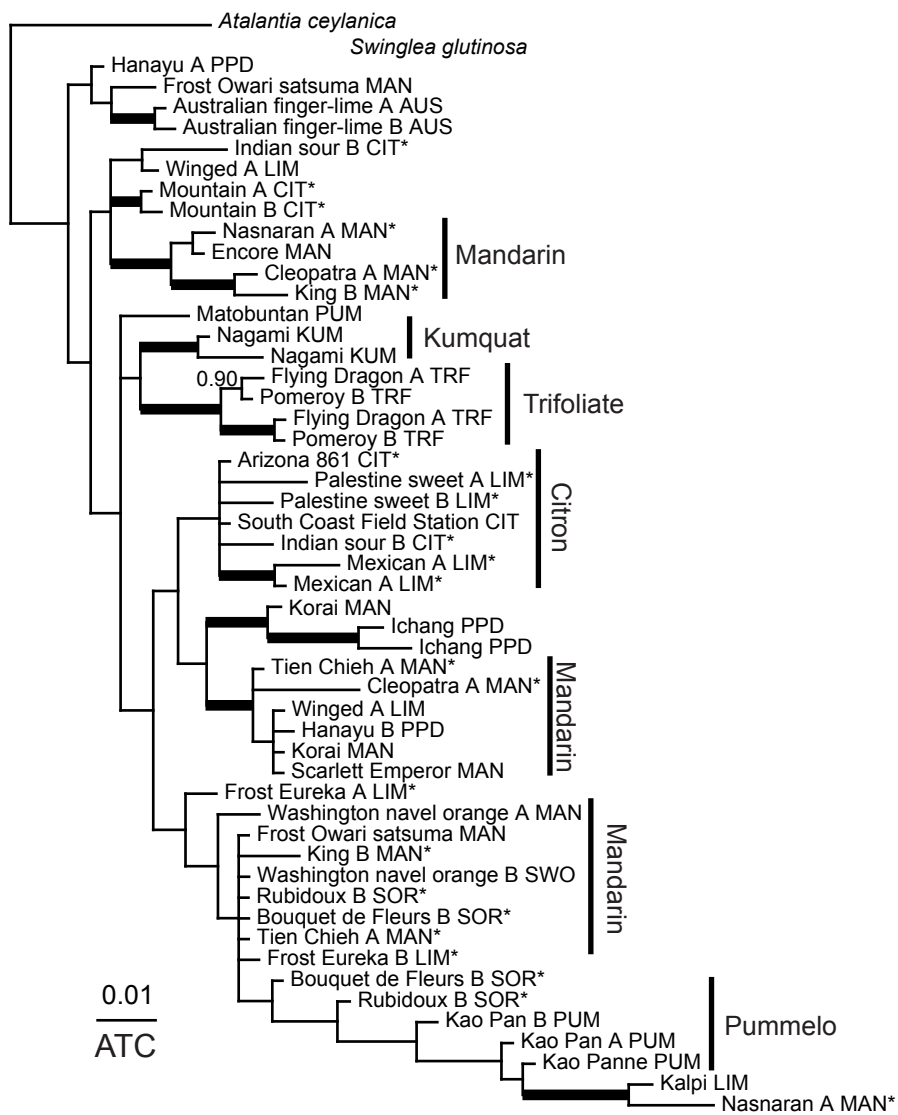

Supplement: Figure S3 — Bayesian consensus phylogram of ATC sequences from Citrus and related genera. Clades with posterior probabilities (PP) of 0.95–1.00 are marked with bold branches; clades with PP less than 0.95 but at least 0.90 are shown above or to the left of branches. Other details as per Figure S2. Groups of alleles discussed in the text are marked by cultivar grouping names. (PDF) [file pone.0068410.s003.pdf]

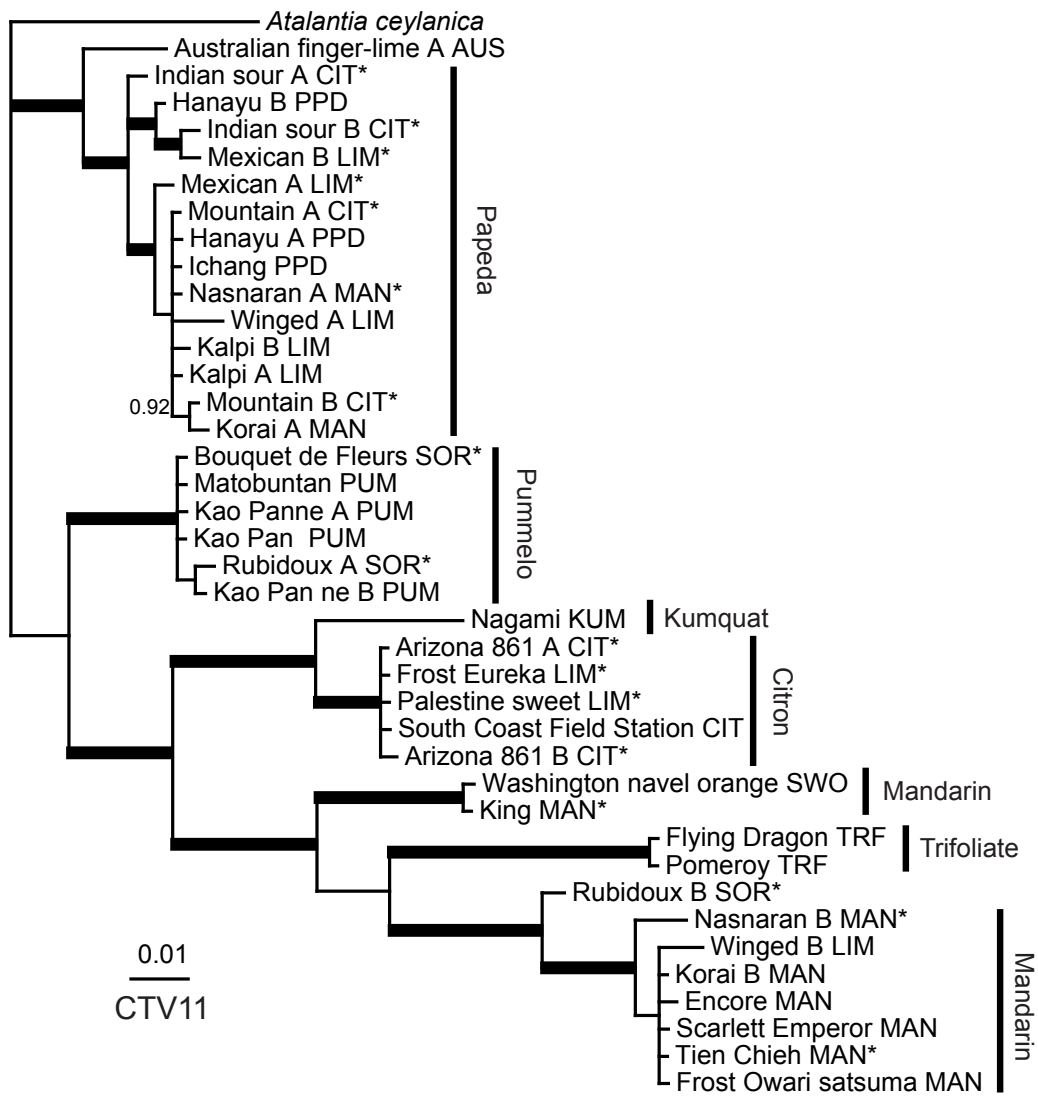

Supplement: Figure S4 — Bayesian consensus phylogram of CTV11 sequences (using all nucleotides) from Citrus and related genera. Clades with posterior probabilities (PP) of 0.95–1.00 are marked with bold branches; clades with PP less than 0.95 but at least 0.90 are shown above or to the left of branches. Other details as per Figure S2. Groups of alleles discussed in the text are marked by cultivar grouping names. (PDF) [file pone.0068410.s004.pdf]

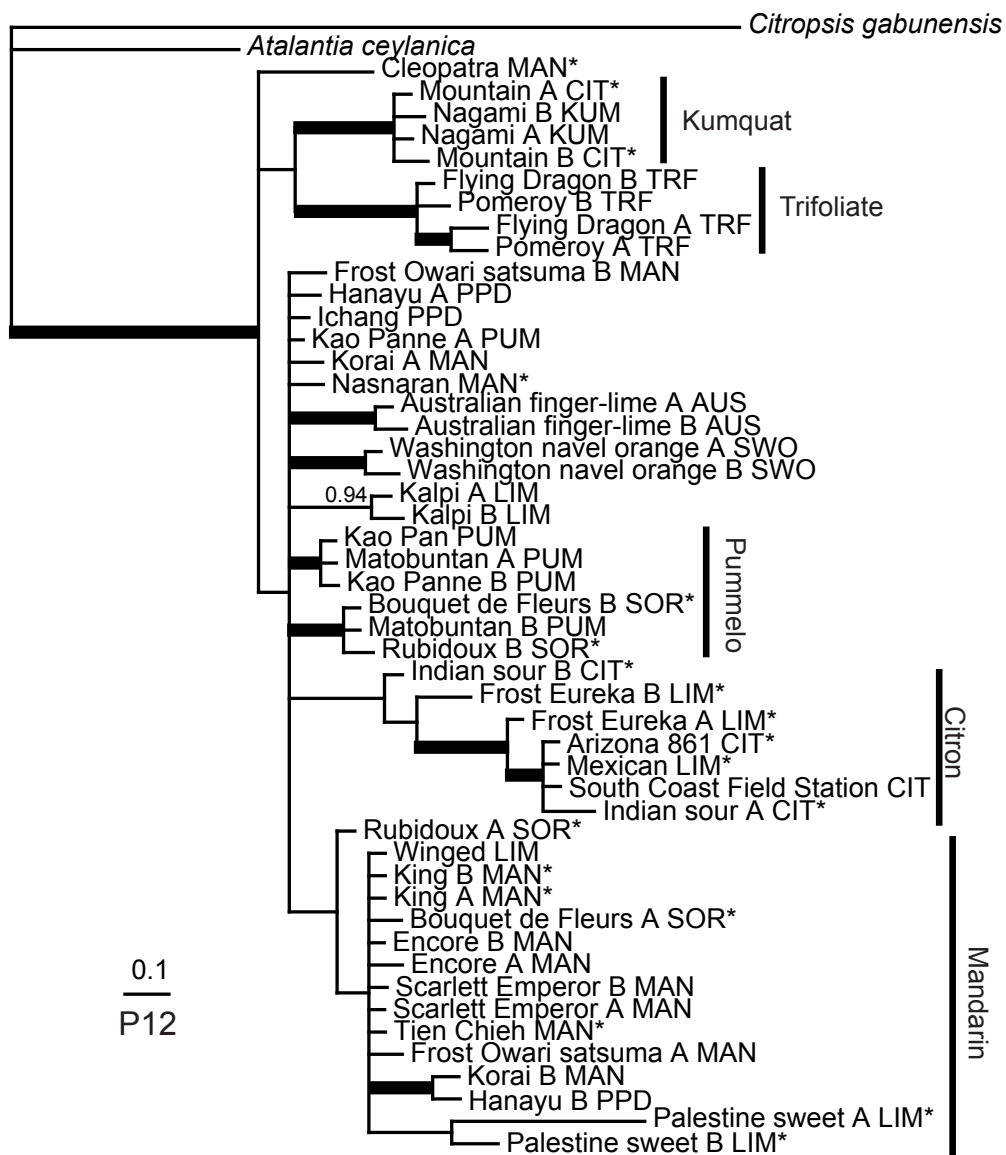

Supplement: Figure S5 — Bayesian consensus phylogram of P12 sequences from Citrus and related genera. Clades with posterior probabilities (PP) of 0.95–1.00 are marked with bold branches; clades with PP less than 0.95 but at least 0.90 are shown above or to the left of branches. Other details as per Figure S2. Groups of alleles discussed in the text are marked by cultivar grouping names. (PDF) [file pone.0068410.s005.pdf]

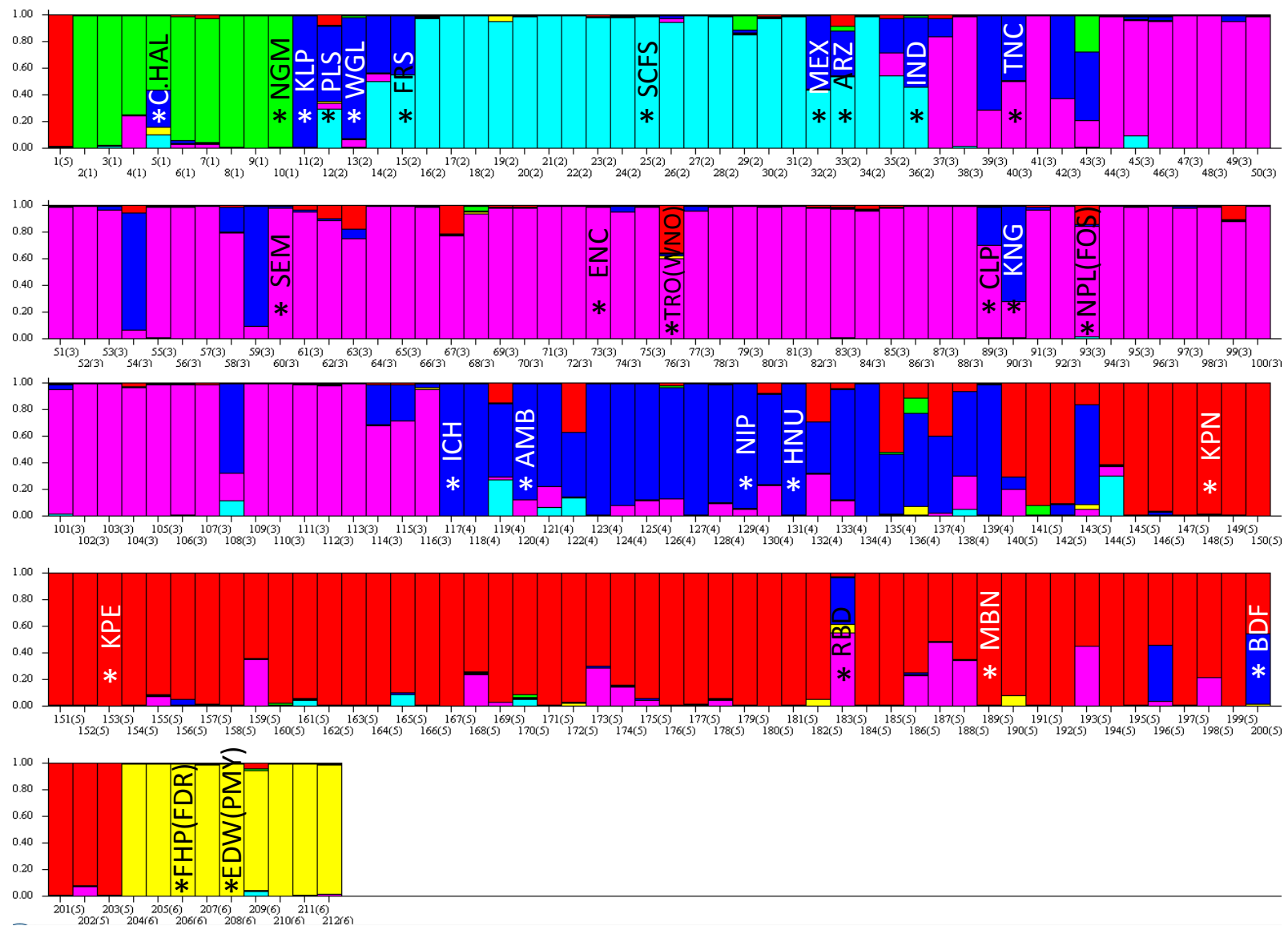

Supplement: Figure S6 — Analysis of 212 Citrus accessions to infer populations by STRUCTURE analysis. Cultivar information is in Table S5. Numbers 2–10 represent Kumquats; 11–36 = limes, lemons and citrons; 37–116 = mandarins; 117–139 = papedas; 140–203 and no. 1 = pummelos; 204–212 = trifoliates. The numbers in parenthesis indicate assumed population groups. Y axis = probable admixture in each accession. Green = kumquat; blue = citron; pink = mandarin; dark blue = papeda; red = pummelo; yellow = trifoliate. One million iterations were run after 500,000 iterations were discarded as “burnin”. Populations assumed, K = 6. Three letter abbreviations are indicated for taxa included in the SNP analysis. Samples 76, 93, 206 and 208 are, Tarocco (TRO), Neopolitana (NPL), Fairhope (FHP) and English Dwarf (EDW) considered equivalent to Washington navel orange (WNO), Frost Owari Satsuma (FOS), Flying Dragon (FDR) and Pomeroy (PMY) respectively. (PDF) [file pone.0068410.s006.pdf]
