# Supplementary material for: A Six Nuclear Gene Phylogeny of Citrus (Rutaceae) Taking into Account Hybridization and Lineage Sorting
Source: PLoS One. 2013 Jul 16;8(7):e68410. doi: 10.1371/journal.pone.0068410 (PMC3713030; doi:10.1371/journal.pone.0068410)
Supplement: Table S1 — Harmonic means of log likelihoods for each model used for each gene for Bayesian analyses based on nruns = 2 and nchains = 10. Model details and the individual trees for each analysis are in Figs 1, 2, and 3 and figures S2, S3, S4. and S5. SD = average standard deviation of split frequencies. Bold likelihoods indicate the model preferred by Bayes factors. (PDF) [file pone.0068410.s007.pdf]

Gene	Model (Partition)	lnL / SD	Model (Partition)	lnL / SD	Model (Partition)	lnL / SD
ATC	HKY + I (DNA), JC (indel)	**-2,793.84** 0.02	GTR + I + G (DNA), JC (indel)	-2,838.14 0.03		
CTVr	HKY + I	**-2,561.22** <0.01	HKY + I + G	-2,648.94 <0.01	GTR + I + G	-2,647.96 0.01
HyB	HKY + I (DNA), JC (indel)	-3,987.50 <0.01	HKY + I (exon), HKY + G (intron), JC (indel)	-4,187.00 <0.01	GTR + I + G (exon), GTR + I + G (intron), JC (indel)	**-3,953.55** 0.01
LGT	HKY + I	**-2,211.10** <0.01	GTR + I + G	-2,269.38 0.01		
MDH	HKY + I (DNA), JC (indel)	-2,485.21 <0.01	HKY + I (exon), HKY + G (intron), JC (indel)	-2,591.15 0.02	GTR + I + G (exon), GTR + I + G (intron), JC (indel)	**-2,460.56** 0.01
P12	HKY + I (DNA), JC (indel)	-2,311.80 0.01	HKY + I (exon), HKY + G (intron), JC (indel)	-2,392.29 0.01	GTR + I + G (exon), GTR + I + G (intron), JC (indel)	**-2,293.66** 0.02
