# Supplementary material for: A Six Nuclear Gene Phylogeny of Citrus (Rutaceae) Taking into Account Hybridization and Lineage Sorting
Source: PLoS One. 2013 Jul 16;8(7):e68410. doi: 10.1371/journal.pone.0068410 (PMC3713030; doi:10.1371/journal.pone.0068410)
Supplement: Table S2 — Inferred allele compositions using Bayesian and parsimony data. Alleles were designated with uppercase letters (in recognized clades with PP>95) or lowercase letters (in clades with PP 80–95). All others were considered to be of unknown origin. Abbreviations used: C = citron; M = mandarin; P = pummelo; D = papeda; K = kumquat; A = Australasian; T = Trifoliate and U = unknown. The four outgroup accessions, Atalantia ceylanica, Swinglea glutinosa, Hesperethusa crenulata and Citropsis gabunensis consisted of unknown alleles. ATC sequences had high homoplasy; allele assignments are less probable than for other genes. (PDF) [file pone.0068410.s008.pdf]

Cultivar/Species	Genes studied						Inferred allele composition
	MDH	P12	ATC[a]	LGT	CTV11	HyB	
‘Arizona 861 S-1’citron	C	C	c	CC	CC	CC	C only
Indian sour citron	CU	Cc	cU	Cc	DD	K	C, D , U and K
‘South Coast Field Station’ citron	C	C	c	C	C	C	C only
Mountain citron	M	KK	UU	UU	DD	K	U, K, D and M
‘Kalpi’ lime	UU	UU	U	U	DD	KK	U, D and K
‘Winged' lime	MU	m	MU	MU	MD	K	M, U, D and K
‘Mexican’ lime	Cc	C	cc	CU	DD	CK	C, D, K, and U
‘Palestine’ sweet lime	CM	mm	cc	C	C	CM	C and M
‘Frost Owari’ satsuma	M	mU	UU	Pp	M		M,U and P
‘Frost Eureka’ lemon	CM	Cc	UU	CM	C	CM	C, M and U
‘Nasnaran' mandarin	MU	U	MU	MU	MD	MK	M, U, D and K.
‘Tien Chieh’ mandarin	MM	m	MU	M	M	MM	Predominantly M
‘Scarlet Emperor’ mandarin	M	mm	M	M	M	MM	M only
‘Encore’ mandarin	MM	mm	M	M	M	MM	M only
‘Korai’ mandarin	M	mU	MD	MM	MD	UM	M, D and U
‘Cleopatra’ mandarin	MM	U	MM	M		MM	Predominantly M
‘Bouquet de Fleurs’ sour orange	MP	mP	Up	M	P	MP	M, P and U
‘Rubidoux’ sour orange	MP	mP	Up	MU	MP	MP	M, P and U
‘Washington' navel orange	MM	UU	UU	M	M	MM	M and U
‘King’ tangor	MM	mm	MU	M	M	MM	Predominantly M
‘Kao Pan’ pummelo	P	P	pp	Pp	P	P	P only
‘Kao Panne’ pummelo	P	PU	p	P	PP	PP	Predominantly P
‘Mato Buntan’ pummelo	P	PP	U	Pp	P	Pp	Predominantly P
'Ichang' papeda	U	U	DD	M	D	DD	D, U and M
‘Hanayu’ papeda	MU	mU	MU	MM	DD		M, U and D
‘Flying dragon’ trifoliate orange	TT	TT	TT	T	T	TT	T only
‘Pomeroy’ trifoliate orange	TT	TT	TT	T	T	TT	T only
‘Nagami’ kumquat	M	KK	KK	U	K	KK	K, M and U
Australian finger-lime	UU	AA	AA	M	A	AA	A, M and U
