# Supplementary material for: A Six Nuclear Gene Phylogeny of Citrus (Rutaceae) Taking into Account Hybridization and Lineage Sorting
Source: PLoS One. 2013 Jul 16;8(7):e68410. doi: 10.1371/journal.pone.0068410 (PMC3713030; doi:10.1371/journal.pone.0068410)
Supplement: Table S3 — Parsimony-based tree characteristics for the six citrus genes studied. (PDF) [file pone.0068410.s009.pdf]

Characteristics	Gene 1: MDH	Gene 2: P12	Gene 3: ATC	Gene 4: LGT	Gene 5: CTVR	Gene 6: HyB
No. of taxa (haplotypes)	51	50	53	44	40	59
Characters analyzed	947	868	863	848	843	1263
Variable characters	129	109	116	114	136	225
Parsimony informative characters	45	53	69	41	108	158
No. of trees	21	35	38	19	12	22
Tree length	164	138	223	146	217	327
Homoplasy index (overall)	0.128	0.137	0.461	0.178	0.294	0.220
Homoplasy index (excluding uninformative characters)	0.283	0.234	0.585	0.366	0.338	0.278
Retention index	0.909	0.904	0.728	0.773	0.927	0.897
Rescaled consistency index	0.793	0.779	0.392	0.572	0.654	0.699
