# Supplementary material for: A Six Nuclear Gene Phylogeny of Citrus (Rutaceae) Taking into Account Hybridization and Lineage Sorting
Source: PLoS One. 2013 Jul 16;8(7):e68410. doi: 10.1371/journal.pone.0068410 (PMC3713030; doi:10.1371/journal.pone.0068410)
Supplement: Table S4 — Difference in tree to tree distance scores between 95% CI of observed gene tree distances from one another and the simulated “gene” tree distances from observed gene trees, under various taxon exclusion sets. *14 putative hybrids (or linegaes of hybrid origin) were removed and compared with 14 randomly removed taxon sets. A p value of <0.05 rejects the null of lineage sorting alone as an explanation for incongruence among observed gene trees. In these cases hybrids remain in the taxon set. The 14 putative hybrids appear to be the smallest set of taxa that can be removed and fail to reject the null, so therefore are the best candidates for having a hybrid origin. Individuals are: 1 ARZ,2 IND,3 SCF,4 HLM,5 KLP,6 WGL,7 MEX,8 PAL,9 FRS,10 AMB,11 TNC,12 SEM,13 ENC,14 NIP,15 CLP,16 BDF,17 RBD,18 WNO,19 KNG,20 KPN,21 KPE,22 MBM,23 ICH,24 FDR,25 PMY,26 NGM,27 MIC. (PDF) [file pone.0068410.s010.pdf]

Taxon Set	95% lower boundary (observed gene to gene distance)	80% critical value upper boundary	Difference	Implied *p* value
None removed	34	11	23	< 0.05
14 Hybrids* BDF FRS HLM ICH IND MEX NGM RBD WGL AMB KLP PAL NIP MIC	5	5	**0**	**> 0.05**
Random1: 17 21 14 13 26 27 18 1 7 4 5 24 19 9	12	4	8	< 0.05
Random2: 13 27 19 16 22 21 20 23 26 17 25 24 9 12	10	4	6	< 0.05
Random3: 24 22 15 23 12 17 26 8 20 18 12 3 27 10	18	4	14	< 0.05
Random4: 8 24 15 22 23 13 18 19 21 3 5 17 25 4	17	4	13	< 0.05
Random5: 12 2 18 6 15 3 9 17 20 4 16 7 27 24	13	4	9	< 0.05
Random6: 3 22 1 7 18 6 19 10 20 15 11 13 12 5	12	4	8	< 0.05
Random7: 15 4 27 12 2 23 13 6 14 21 20 18 17 26	10	3	7	< 0.05
Random8: 7 12 21 6 22 5 15 9 10 25 14 19 23 4	13	4	9	< 0.05
Random9: 20 4 21 18 15 17 1 14 6 22 16 9 5 8	10	4	6	< 0.05
Random10: 23 26 17 20 15 7 8 18 14 21 13 22 6 11	11	4	7	< 0.05
