# Supplementary material for: A Six Nuclear Gene Phylogeny of Citrus (Rutaceae) Taking into Account Hybridization and Lineage Sorting
Source: PLoS One. 2013 Jul 16;8(7):e68410. doi: 10.1371/journal.pone.0068410 (PMC3713030; doi:10.1371/journal.pone.0068410)
Supplement: Table S5 — Accessions included in the STRUCTURE analysis. For the SSR analysis, we included a subset of 212 accessions from a total of 370 accessions reported by Barkley et al (2006). Many hybrids were excluded from the analysis. Accessions with an asterisk (*) were also included in the SNP analysis. Accessions with two asterisks (**) were selected as being very similar to Parent Washington navel orange (CRC 3596, Tarocco), Frost Owari Satsuma (CRC 3848, Neopolitana), Flying Dragon (CRC 3351, Fairhope) and Pomeroy trifoliate (CRC 3876, English Dwarf). (PDF) [file pone.0068410.s011.pdf]

| No. | CRC no. | Cultivar name | Group | Genus | Species (Swingle) | Species (Tanaka) |
|---|---|---|---|---|---|---|
| 1 | 1208 | Roedings Pink | Pummelo | *Citrus* | *maxima* | *maxima* |
| 2 | 1471 | Meiwa | Kumquat | *Fortunella* | *crassifolia* | *crassifolia* |
| 3 | 3237 | *Fortunella japonica* | Kumquat | *Fortunella* | *japonica* | *japonica* |
| 4 | 3475 | Changshou | Kumquat | *Fortunella* | *obovata* | *obovata* |
| 5 | 3780 | *Citrus halimii** | Citron hybrid | *Citrus* | N/A | *halimii* |
| 6 | 3789 | Unnamed | Kumquat | *Fortunella* | *hindsii* | *hindsii* |
| 7 | 3790 | BB394 | Kumquat | *Fortunella* | *hindsii* | *hindsii* |
| 8 | 3818 | Meiwa | Kumquat | *Fortunella* | *crassifolia* | *crassifolia* |
| 9 | 3833 | Meiwa | Kumquat | *Fortunella* | *crassifolia* | *crassifolia* |
| 10 | 3877 | Nagami* | Kumquat | *Fortunella* | *margarita* | *margarita* |
| 11 | 1455 | Kalpi* | Lime | *Citrus* | *aurantifolia* | *webberi* |
| 12 | 1482 | Palestine* | Sweet lime | *Citrus* | *aurantifolia* | *limettoides* |
| 13 | 2320 | Winged Lime* | Lime | *Citrus* | *aurantifolia* | *longispina* |
| 14 | 2424 | Borneo Rangpur | Rangpur | *Citrus* | *limon* | *limonia* |
| 15 | 3005 | Frost Eureka* | Lemon Eureka | *Citrus* | *limon* | *limon* |
| 16 | 3518 | Citron of Commerce | Citron | *Citrus* | *medica* | *medica* |
| 17 | 3523 | Diamante | Citron | *Citrus* | *medica* | *medica* |
| 18 | 3527 | Hiawassie | Citron | *Citrus* | *medica* | *medica* |
| 19 | 3530 | Italian | Citron | *Citrus* | *medica* | *medica* |
| 20 | 3531 | Mexican | Citron | *Citrus* | *medica* | *medica* |
| 21 | 3532 | Papuan | Citron | *Citrus* | *medica* | *medica* |
| 22 | 3533 | Philippine | Citron | *Citrus* | *medica* | *medica* |
| 23 | 3534 | Sicily | Citron | *Citrus* | *medica* | *medica* |
| 24 | 3535 | Spadifora | Citron | *Citrus* | *medica* | *medica* |
| 25 | 3546 | South Coast Field Station* | Citron | *Citrus* | *medica* | *medica* |
| 26 | 3593 | Interdonato | Citron | *Citrus* | *medica* | *medica* |
| 27 | 3654 | Dulcia | Citron | *Citrus* | *medica* | *medica* |
| 28 | 3655 | Odorata | Citron | *Citrus* | *medica* | *medica* |
| 29 | 3768 | Buddha's hand | Citron | *Citrus* | *medica* | *medica* |
| 30 | 3798 | Yunnanensis | Citron | *Citrus* | *medica* | *medica* |
| 31 | 3819 | Unnamed | Citron | *Citrus* | *medica* | *medica* |
| 32 | 3822 | Mexican* | Lime | *Citrus* | *medica* | *medica* |
| 33 | 3878 | Arizona S1* | Citron | *Citrus* | N/A | *medica* |
| 34 | 3891 | Ethrog | Citron | *Citrus* | *medica* | *medica* |
| 35 | 569 | Millsweet | Sweet lemon | *Citrus* | *limon* | *limetta* |
| 36 | 661 | Indian* | Citron | *Citrus* | *medica* | *medica* |
| 37 | 2331 | HungKat | Mandarin | *Citrus* | *reticulata* | *reticulata* |
| 38 | 2376 | Tien Chieh | Mandarin | *Citrus* | *reticulata* | *reticulata* |
| 39 | 2448 | *C. depressa* | Mandarin | *Citrus* | *reticulata* | *depressa* |
| 40 | 2590 | Tien Chieh* | Mandarin | *Citrus* | *reticulata* | *reticulata* |
| 41 | 2692 | Timkat | Mandarin | *Citrus* | *reticulata* | *oleocarpa* |
| 42 | 2710 | *C. depressa* | Mandarin | *Citrus* | *reticulata* | *depressa* |
| 43 | 2867 | Calashu | Mandarin | *Citrus* | *reticulata* | *hybrid* |
| 44 | 2893 | Laranja Cravo | Mandarin | *Citrus* | *reticulata* | *reticulata* |
| 45 | 300 | Parson's Special | Mandarin | *Citrus* | *reticulata* | *reticulata* |
| 46 | 3019 | Kara | Mandarin | *Citrus* | *reticulata* | *reticulata* |
| 47 | 3020 | Wilking | Mandarin | *Citrus* | *reticulata* | *reticulata* |
| 48 | 3021 | Kinnow | Mandarin | *Citrus* | *reticulata* | *reticulata* |
| 49 | 3022 | Frua | Mandarin | *Citrus* | *reticulata* | *reticulata* |
| 50 | 3026 | Dancy (Frost nucellar) | Mandarin | *Citrus* | *reticulata* | *tangerina* |
| 51 | 303 | King | Mandarin | *Citrus* | *reticulata* | *nobilis* |

| | | | | | | |
|---|---|---|---|---|---|---|
| 52 | 3085 | Szinkom | Mandarin | *Citrus* | *reticulata* | *reticulata* |
| 53 | 3143 | *C. sunki* | Mandarin | *Citrus* | *reticulata* | *sunki* |
| 54 | 3147 | *C. leiocarpa* | Mandarin | *Citrus* | *reticulata* | *leiocarpa* |
| 55 | 3177 | Honey | Mandarin | *Citrus* | *reticulata* | *reticulata* |
| 56 | 3239 | Unnamed | Mandarin | *Citrus* | *reticulata* | *reticulata* |
| 57 | 3260 | SohNiamtra | Mandarin | *Citrus* | *reticulata* | *reticulata* |
| 58 | 3292 | Fukushu | Mandarin | *Citrus* | *reticulata* | *erythrosa* |
| 59 | 3297 | *C. tardiva* | Mandarin | *Citrus* | *reticulata* | *tardiva* |
| 60 | 3326 | ScarletEmperor* | Mandarin | *Citrus* | *reticulata* | *reticulata* |
| 61 | 3328 | SolidScarlet | Mandarin | *Citrus* | *reticulata* | *reticulata* |
| 62 | 3329 | Richards Special | Mandarin | *Citrus* | *reticulata* | *reticulata* |
| 63 | 3346 | Kunembo | Mandarin | *Citrus* | *reticulata* | *nobilis* |
| 64 | 3363 | Belady | Mandarin | *Citrus* | *reticulata* | *reticulata* |
| 65 | 3367 | Mandarine Sanguine | Mandarin | *Citrus* | *reticulata* | *reticulata* |
| 66 | 3405 | Mandarinette | Mandarin | *Citrus* | *reticulata* | *reticulata* |
| 67 | 3466 | *C. yatsushiro* | Mandarin | *Citrus* | *reticulata* | *yatsushiro* |
| 68 | 3558 | Fremont | Mandarin | *Citrus* | *reticulata* | *reticulata* |
| 69 | 3559 | Fairchild | Mandarin | *Citrus* | *reticulata* | *reticulata* |
| 70 | 3560 | Fortune | Mandarin | *Citrus* | *reticulata* | *reticulata* |
| 71 | 3564 | *C. lycopersicaeformis* | Mandarin | *Citrus* | *reticulata* | *lycopersicaeformis* |
| 72 | 3568 | Pixie | Mandarin | *Citrus* | *reticulata* | *reticulata* |
| 73 | 3569 | Encore* | Mandarin | *Citrus* | *reticulata* | *reticulata* |
| 74 | 3576 | Canton | Mandarin | *Citrus* | *reticulata* | *reticulata* |
| 75 | 3577 | Changsha | Mandarin | *Citrus* | *reticulata* | *reticulata* |
| 76 | 3596 | Tarocco ** | Blood orange | *Citrus* | *sinensis* | *sinensis* |
| 77 | 3613 | Empress | Mandarin | *Citrus* | *reticulata* | *reticulata* |
| 78 | 3615 | Nova | Mandarin | *Citrus* | *reticulata* | *reticulata* |
| 79 | 3649 | Bower | Mandarin | *Citrus* | *reticulata* | *reticulata* |
| 80 | 3659 | Batangas | Mandarin | *Citrus* | *reticulata* | *reticulata* |
| 81 | 3727 | Nagpur | Mandarin | *Citrus* | *reticulata* | *reticulata* |
| 82 | 3731 | Unnamed | Mandarin | *Citrus* | *reticulata* | *reticulata* |
| 83 | 3738 | Unnamed | Mandarin | *Citrus* | *reticulata* | *reticulata* |
| 84 | 3752 | SomKeowan | Mandarin | *Citrus* | *reticulata* | *reticulata* |
| 85 | 3809 | Sunburst | Mandarin | *Citrus* | *reticulata* | *reticulata* |
| 86 | 3812 | Unnamed | Mandarin | *Citrus* | *reticulata* | *reticulata* |
| 87 | 3813 | Unnamed | Mandarin | *Citrus* | *reticulata* | *reticulata* |
| 88 | 3843 | Willowleaf | Mandarin | *Citrus* | *reticulata* | *deliciosa* |
| 89 | 3844 | Cleopatra* | Mandarin | *Citrus* | *reticulata* | *reshni* |
| 90 | 3845 | King* | Mandarin | *Citrus* | *reticulata* | *nobilis* |
| 91 | 3846 | Murcott | Mandarin | *Citrus* | *reticulata* | *reticulata* |
| 92 | 3847 | Hill | Mandarin | *Citrus* | *reticulata* | *reticulata* |
| 93 | 3848 | Neopolitana** | Mandarin | *Citrus* | *reticulata* | *unshiu* |
| 94 | 3849 | Ponkan | Mandarin | *Citrus* | *reticulata* | *reticulata* |
| 95 | 3850 | Robinson | Mandarin | *Citrus* | *reticulata* | *reticulata* |
| 96 | 3851 | Lee | Mandarin | *Citrus* | *reticulata* | *reticulata* |
| 97 | 3852 | Som Kaeo II | Mandarin | *Citrus* | *reticulata* | *reticulata* |
| 98 | 3853 | Sam Saa | Mandarin | *Citrus* | *reticulata* | *reticulata* |
| 99 | 3880 | *C. yatsushiro* | Mandarin | *Citrus* | *reticulata* | *yatsushiro* |
| 100 | 3887 | Kinokuni | Mandarin | *Citrus* | *reticulata* | *kinokuni* |
| 101 | 3895 | Unnamed | Mandarin | *Citrus* | *reticulata* | *reticulata* |

| | | | | | | |
|---|---|---|---|---|---|---|
| 102 | 3897 | Huang Yen Man Chieh | Mandarin | *Citrus* | *reticulata* | *reticulata* |
| 103 | 3906 | Seedless Kishu | Mandarin | *Citrus* | *reticulata* | *reticulata* |
| 104 | 3910 | Daisy | Mandarin | *Citrus* | *reticulata* | *reticulata* |
| 105 | 3913 | Unnamed | Mandarin | *Citrus* | *reticulata* | *reticulata* |
| 106 | 3953 | W.Murcott | Mandarin | *Citrus* | *reticulata* | *reticulata* |
| 107 | 3958 | Koster | Mandarin | *Citrus* | *reticulata* | *reticulata* |
| 108 | 3965 | NISSV E | Mandarin | *Citrus* | *reticulata* | *hybrid* |
| 109 | 3990 | Fallglo | Mandarin | *Citrus* | *reticulata* | *reticulata* |
| 110 | 3991 | Unnamed (USDA 88-2) | Mandarin | *Citrus* | *reticulata* | *reticulata* |
| 111 | 3992 | Unnamed | Mandarin | *Citrus* | *reticulata* | *reticulata* |
| 112 | 4003 | Sun Chu Sha | Mandarin | *Citrus* | *reticulata* | *reticulata* |
| 113 | 4011 | Clementina Fina | Mandarin | *Citrus* | *reticulata* | *clementina* |
| 114 | 4021 | Temple X Dancy | Mandarin | *Citrus* | *reticulata* | *reticulata* |
| 115 | 4031 | Rubidoux | Mandarin | *Citrus* | *reticulata* | *reticulata* |
| 116 | 696 | Kinokuni | Mandarin | *Citrus* | *tachibana* | *kinokuni* |
| 117 | 2327 | *C. ichangensis** | Papeda | *Citrus* | *ichangensis* | *ichangensis* |
| 118 | 2431 | *C. ichangensis* | Papeda | *Citrus* | *ichangensis* | *ichangensis* |
| 119 | 2454 | *C. hystrix* | Papeda | *Citrus* | *hystrix* | *hystrix* |
| 120 | 2485 | *C. amblycarpa** | Mandarin | *Citrus* | *reticulata* | *amblycarpa* |
| 121 | 3052 | *C. latipes* | Papeda | *Citrus* | *latipes* | *latipes* |
| 122 | 3056 | Unnamed | Papeda | *Citrus* | *species* | *species* |
| 123 | 3103 | *C. hystrix* | Papeda | *Citrus* | *hystrix* | *hystrix* |
| 124 | 3144 | *C. keraji* | Mandarin | *Citrus* | *reticulata* | *keraji* |
| 125 | 3150 | *C. tachibana* | Mandarin | *Citrus* | *tachibana* | *tachibana* |
| 126 | 3175 | Unnamed | Sour Orange Hybrid | *Citrus* | *species* | *species* |
| 127 | 3203 | Soh Niangrang | Papeda | *Citrus* | *species* | *species* |
| 128 | 3225 | *C. maderaspatana* | Sour Orange Hybrid | *Citrus* | *aurantium* | *maderaspatana* |
| 129 | 3228 | *C. nippokoreana** | Mandarin | *Citrus* | *reticulata* | *nippokoreana* |
| 130 | 3280 | *C. succosa* | Mandarin | *Citrus* | *reticulata* | *succosa* |
| 131 | 3469 | Hanayu* | Papeda | *Citrus* | N/A | *hanaju* |
| 132 | 3474 | *C. intermedia* | Sour Orange Hybrid | *Citrus* | *aurantium hybrid* | *intermedia* |
| 133 | 3574 | *C. miaray* | Sour Orange Hybrid | *Citrus* | *hybrid* | *miaray* |
| 134 | 3605 | Microcarpa | Papeda | *Citrus* | *micrantha* | *micrantha* |
| 135 | 3793 | Unnamed | Papeda | *Oxanthera* | *neo-caledonica* | *neo-caledonica* |
| 136 | 3797 | Unnamed | Papeda | *Citrus* | *species* | *hongensis* |
| 137 | 3931 | *C. ichangensis* | Papeda | *Citrus* | *ichangensis* | *ichangensis* |
| 138 | 3943 | Kabosu | Papeda | *Citrus* | *species* | *sphaerocarpa* |
| 139 | 432 | Cabuyao | Papeda | *Citrus* | *hystrix* | *hystrix* |
| 140 | 1212 | Unnamed | Pummelo | *Citrus* | *maxima* | *maxima* |
| 141 | 1224 | Unnamed | Pummelo | *Citrus* | *maxima* | *maxima* |
| 142 | 1225 | Hunnan | Pummelo | *Citrus* | *maxima* | *maxima* |
| 143 | 1689 | Brazilian | Sour Orange | *Citrus* | *aurantium* | *aurantium* |
| 144 | 1775 | Lemon Shaddock | Pummelo hybrid | *Citrus* | *maxima hybrid* | *maxima* |
| 145 | 2236 | Sunshine | Pummelo | *Citrus* | *maxima* | *maxima* |
| 146 | 2240 | Siamese Acidless | Pummelo | *Citrus* | *maxima* | *maxima* |
| 147 | 2241 | Kao Pan | Pummelo | *Citrus* | *maxima* | *maxima* |
| 148 | 2242 | Kao Pan* | Pummelo | *Citrus* | *maxima* | *maxima* |
| 149 | 2243 | Kao Pan | Pummelo | *Citrus* | *maxima* | *maxima* |
| 150 | 2244 | Pink | Pummelo | *Citrus* | *maxima* | *maxima* |
| 151 | 2245 | Red | Pummelo | *Citrus* | *maxima* | *maxima* |
| 152 | 2246 | Pink | Pummelo | *Citrus* | *maxima* | *maxima* |
| 153 | 2248 | Kao Panne* | Pummelo | *Citrus* | *maxima* | *maxima* |

| | | | | | | |
|---|---|---|---|---|---|---|
| 154 | 2249 | Kao Panne | Pummelo | *Citrus* | *maxima* | *maxima* |
| 155 | 2338 | Unnamed | Pummelo | *Citrus* | *maxima* | *maxima* |
| 156 | 2340 | Unnamed | Pummelo | *Citrus* | *maxima* | *maxima* |
| 157 | 2341 | KarnLauYau | Pummelo | *Citrus* | *maxima* | *maxima* |
| 158 | 2342 | PongYau | Pummelo | *Citrus* | *maxima* | *maxima* |
| 159 | 2343 | Philippine | Pummelo | *Citrus* | *maxima* | *maxima* |
| 160 | 2346 | African | Pummelo | *Citrus* | *maxima* | *maxima* |
| 161 | 2347 | DeepRed | Pummelo | *Citrus* | *maxima* | *maxima* |
| 162 | 2348 | Pin Shan Kong Yau | Pummelo | *Citrus* | *maxima* | *maxima* |
| 163 | 2349 | Kao Panne | Pummelo | *Citrus* | *maxima* | *maxima* |
| 164 | 2350 | Kao Ruan Tia | Pummelo | *Citrus* | *maxima* | *maxima* |
| 165 | 2351 | Kao Ruan Tia | Pummelo | *Citrus* | *maxima* | *maxima* |
| 166 | 2352 | Kao Phuang | Pummelo | *Citrus* | *maxima* | *maxima* |
| 167 | 2353 | Nakhon Chaisi | Pummelo | *Citrus* | *maxima* | *maxima* |
| 168 | 2355 | Kao Panne | Pummelo | *Citrus* | *maxima* | *maxima* |
| 169 | 2356 | Kao Panne | Pummelo | *Citrus* | *maxima* | *maxima* |
| 170 | 2421 | Siamese | Pummelo | *Citrus* | *maxima* | *maxima* |
| 171 | 2453 | Unnamed | Pummelo | *Citrus* | *maxima* | *maxima* |
| 172 | 2487 | Alemoen | Pummelo | *Citrus* | *maxima* | *maxima* |
| 173 | 2583 | Tau Yau | Pummelo | *Citrus* | *maxima* | *maxima* |
| 174 | 2596 | Arajon | Pummelo | *Citrus* | *maxima* | *maxima* |
| 175 | 2608 | Red Aranyan | Pummelo | *Citrus* | *maxima* | *maxima* |
| 176 | 2752 | PanDan | Pummelo | *Citrus* | *maxima* | *maxima* |
| 177 | 3067 | Sweet | Pummelo | *Citrus* | *maxima* | *maxima* |
| 178 | 3148 | *C. sinograndis* | Pummelo | *Citrus* | *maxima* | *sinograndis* |
| 179 | 3224 | Chandler | Pummelo | *Citrus* | *maxima* | *maxima* |
| 180 | 3282 | Unnamed | Pummelo | *Citrus* | *maxima* | *maxima* |
| 181 | 3805 | Reinking | Pummelo | *Citrus* | *maxima* | *maxima* |
| 182 | 3806 | Tahitian | Pummelo | *Citrus* | *maxima* | *maxima* |
| 183 | 3855 | Rubidoux* | Sour Orange | *Citrus* | *aurantium* | *aurantium* |
| 184 | 3926 | Kao Phuang | Pummelo | *Citrus* | *maxima* | *maxima* |
| 185 | 3927 | Thong Dee | Pummelo | *Citrus* | *maxima* | *maxima* |
| 186 | 3928 | Itoshima Bankan | Pummelo | *Citrus* | *maxima* | *maxima* |
| 187 | 3940 | HaikuB | Pummelo | *Citrus* | *maxima* | *maxima* |
| 188 | 3944 | Kawachi-Bankan | Pummelo | *Citrus* | *maxima* | *maxima* |
| 189 | 3945 | Mato Buntan* | Pummelo | *Citrus* | *maxima* | *maxima* |
| 190 | 3947 | Suisho Buntan | Pummelo | *Citrus* | *maxima* | *maxima* |
| 191 | 3948 | Kao Pan | Pummelo | *Citrus* | *maxima* | *maxima* |
| 192 | 3949 | Pauthel | Pummelo | *Citrus* | *maxima* | *maxima* |
| 193 | 3950 | Banokan | Pummelo | *Citrus* | *maxima* | *maxima* |
| 194 | 3959 | Egami Buntan | Pummelo | *Citrus* | *maxima* | *maxima* |
| 195 | 3961 | Banpeiyu | Pummelo | *Citrus* | *maxima* | *maxima* |
| 196 | 3979 | Anseikan | Pummelo | *Citrus* | *species* | *species* |
| 197 | 4026 | Pomelit | Pummelo | *Citrus* | *maxima* | *maxima* |
| 198 | 4028 | Rubidoux | Pummelo | *Citrus* | *maxima* | *maxima* |
| 199 | 448 | Moanalua | Pummelo | *Citrus* | *maxima* | *maxima* |
| 200 | 571 | Bouquet des Fleurs* | Sour Orange | *Citrus* | *aurantium* | *aurantium* |
| 201 | 578 | Fleming's Shaddock | Pummelo | *Citrus* | *maxima* | *maxima* |
| 202 | 640 | Siamese Seedless | Pummelo | *Citrus* | *maxima* | *maxima* |

| | | | | | | |
|---|---|---|---|---|---|---|
| 203 | 644 | Philippine | Pummelo | *Citrus* | *maxima* | *maxima* |
| 204 | 2554 | Barnes | Trifoliate | *Poncirus* | *trifoliata* | *trifoliata* |
| 205 | 3151 | Australian | Trifoliate | *Poncirus* | *trifoliata* | *trifoliata* |
| 206 | 3351 | Fairhope ** | Trifoliate | *Poncirus* | *trifoliata* | *trifoliata* |
| 207 | 3549 | Simmons | Trifoliate | *Poncirus* | *trifoliata* | *trifoliata* |
| 208 | 3876 | English Dwarf ** | Trifoliate | *Poncirus* | *trifoliata* | *trifoliata* |
| 209 | 3888 | Unnamed | Trifoliate | *Poncirus* | *trifoliata* | *trifoliata* |
| 210 | 3938 | #27 China | Trifoliate | *Poncirus* | *trifoliata* | *trifoliata* |
| 211 | 4007 | Seedling | Trifoliate | *Poncirus* | *trifoliata* | *trifoliata* |
| 212 | 4008 | Seedling | Trifoliate | *Poncirus* | *trifoliata* | *trifoliata* |
