# Supplementary material for: A Six Nuclear Gene Phylogeny of Citrus (Rutaceae) Taking into Account Hybridization and Lineage Sorting
Source: PLoS One. 2013 Jul 16;8(7):e68410. doi: 10.1371/journal.pone.0068410 (PMC3713030; doi:10.1371/journal.pone.0068410)
Supplement: Table S6 — Inferred ancestry of individuals included in Structure and SNP analysis. Numbers in columns 5–11 represent proportion of alleles scored based on STRUCTURE and SNP values (in parenthesis). a Represents the identifying numbers used in Structure analysis (Fig S6). b Citrus variety collection reference numbers. c Tentative groups recognized by Barkley et al [21]. Other abbreviations used: PUM = pummelo; KUM = kumquat; PPD = papeda; TRF = trifoliate; MAN = mandarin; CIT = citron. Column designated “other” refers to alleles that were not assigned to any of the other recognized groups in SNP analysis. *Accessions with more than 10% admixture by STRUCTURE. # Hybrids according to coalescence analysis but not by STRUCTURE analysis. (PDF) [file pone.0068410.s012.pdf]

Inferred ancestry of individuals included in Structure and SNP analysis

No.[a]	CRC no.[b]	Cultivar name	Gp[c]	PUM(P)	KUM(K)	PPD(D)	TRF(T)	MAN(M)	CIT(C)	Other(O)
5	3780	Mountain citron*	1	0.002(0)	0.556(0.33)	0.28(0.17)	0.058(0)	0.002(0.17)	0.102(0)	(0.33)
10	3877	Nagami#	1	0.001(0)	0.985(0.67)	0.005(0)	0.002(0)	0.004(0.17)	0.002(0)	(0.16)
11	1455	Kalpi#	2	0.002(0)	0.004(0.17)	0.982(0.17)	0.001(0)	0.002(0)	0.008(0)	(0.66)
12	1482	Palestine*	2	0.078(0)	0.007(0)	0.559(0)	0.02(0)	0.042(0.33)	0.294(0.5)	(0.17)
13	2320	Winged lime	2	0.006(0)	0.009(0.17)	0.911(0.08)	0.004(0)	0.056(0.5)	0.014(0)	(0.25)
15	3005	Frost Eureka*	2	0.003(0)	0.004(0)	0.438(0)	0.001(0)	0.003(0.25)	0.551(0.5)	(0.25)
25	3546	South Coast Field Station	2	0.001(0)	0.003(0)	0.001(0)	0.001(0)	0.002(0)	0.992(0.83)	(0.17)
32	3822	Mexican*	2	0.001(0)	0.004(0.08)	0.549(0.17)	0.005(0)	0.001(0)	0.44(0.58)	(0.17)
33	3878	Arizona 861 S-1*	2	0.079(0)	0.042(0)	0.333(0)	0.006(0)	0.003(0)	0.537(0.83)	(0.17)
36	661	Indian*	2	0.002(0)	0.014(0.17)	0.52(0.17)	0.003(0)	0.002(0)	0.458(0.25)	(0.5)
40	2590	Tien Chieh*	3	0.002(0)	0.002(0)	0.487(0)	0.002(0)	0.506(0.75)	0.001(0)	(0.25)
60	3326	Scarlett Emperor	3	0.001(0)	0.006(0)	0.008(0)	0.001(0)	0.982(0.83)	0.001(0)	(0.17)
73	3569	Encore	3	0.003(0)	0.001(0)	0.003(0)	0.001(0)	0.991(0.83)	0.002(0)	(0.17)
89	3844	Cleopatra*	3	0.001(0)	0.007(0)	0.29(0)	0.001(0)	0.69(0.8)	0.011(0)	(0.2)
90	3845	King*	3	0.003(0)	0.002(0)	0.711(0)	0.001(0)	0.275(0.75)	0.008(0)	(0.25)
117	2327	Ichang#	4	0.002(0)	0.005(0)	0.989(50)	0.001(0)	0.002(0.17)	0.001(0)	(0.33)
120	2485	Nasnaran*	4	0.001(0)	0.003(0.08)	0.871(0.08)	0.001(0)	0.122(0.5)	0.002(0)	(0.33)
129	3228	Korai	4	0.004(0)	0.002(0)	0.935(0.17)	0.003(0)	0.055(0.58)	0.002(0)	(0.25)
131	3469	Hanayu	4	0.001(0)	0.002(0)	0.985(0.2)	0.005(0)	0.003(0.5)	0.003(0)	(0.3)
148	2242	Kao Pan	5	0.985(0.75)	0.002(0)	0.002(0)	0.001(0)	0.003(0)	0.006(0)	(0.25)
153	2248	Kao Panne	5	0.993(0.75)	0.001(0)	0.002(0)	0.001(0)	0.003(0)	0.001(0)	(0.25)
183	3855	Rubidoux*	5	0.028(0.33)	0.001(0)	0.354(0)	0.064(0)	0.547(0.33)	0.006(0)	(0.33)
189	3945	Mato Buntan	5	0.99(0.67)	0.002(0)	0.002(0)	0.001(0)	0.003(0)	0.001(0)	(0.33)
200	571	Bouquet des Fleurs*	5	0.452(0.42)	0.002(0)	0.525(0)	0.014(0)	0.003(0.33)	0.002(0)	(0.25)
