# Supplementary material for: A Six Nuclear Gene Phylogeny of Citrus (Rutaceae) Taking into Account Hybridization and Lineage Sorting
Source: PLoS One. 2013 Jul 16;8(7):e68410. doi: 10.1371/journal.pone.0068410 (PMC3713030; doi:10.1371/journal.pone.0068410)
Supplement: Appendix S1 — Methods used for coalescent simulation analyses are described in detail. (DOC) [file pone.0068410.s013.doc]

**Appendix**

Methods

Briefly, the coalescent simulation analysis to detect hybridization apart from lineage sorting was done as follows: (1) gene trees were inferred in Mr Bayes using allelic data; (2) a pool of trees from the stable posterior distribution were converted to chronograms (phylogenetic trees depicting evolutionary time through branch lengths) in r8s; (3) the chronograms were scaled to absolute time in Mesquite using previous date estimates and mutation rates were derived for each locus ; (4) allelic diversity (θ) was estimated for each locus with the sequence data using MCCoal; from this and the mutation rate, the effective population size (*N_e_*) was derived using either θ = 4.µ.*N_e_* or θ = 2.µ.*N_e_* depending on the breeding system; (5) using the chronograms, *N_e_* and generation times for the species, we simulated under the coalescent new “gene trees” using Mesquite; (6) we generated a distribution of tree-to-tree distances (symmetric distance in PAUP*) for each observed gene tree and corresponding simulated trees, as well as among the gene trees; (7) we compared these distributions to determine whether significant differences exist among the observed gene trees in light of an incomplete lineage sorting null hypothesis.

Additional details for each step are now described. For step 1, we used the three most resolved and recombination-free gene trees (LGT, MDH and HYB – see Results). For step 2, we drew 20 trees from the end of the posterior distribution in order to account for uncertainty in our gene tree estimation. These trees are topologically representative of the complete Bayesian analyses (BA) for each locus (consensus topologies shown in Figures S1A,S1B and S1C) and are representative with regard to tree-to-tree distance of each locus from each other, producing approximately normal distributions (Figure 4). Conversion to chronograms used r8s version 1.71 (Sanderson, 2003) and was done using the penalized likelihood function (Sanderson, 2002) so that the branch lengths estimate evolutionary time in a gene tree. The smoothing value (method to simultaneously estimate unknown divergence times and smooth the rapidity of change along lineages) was assessed between 10^-3^ to 10^3^ for every 0.3 power increment, with the locus-appropriate value used in each case for the final analysis. Each chronogram was then trimmed to contain only a single allele from each individual, using Mesquite (Maddison and Maddison, 2006) to delete terminals while maintaining appropriate branch lengths. The remaining alleles were chosen to *maximize* topological incongruence among the loci, because several loci with alleles in the same clade do not introduce incongruence beyond incomplete lineage sorting and thus require no further explanation.

For step 3, the branch lengths were scaled in Mesquite such that branch lengths were in units of generations, based on a calibration point of the divergence between *Poncirus trifoliata* and *Citrus sinensis* of 5.9 Ma (Pfeil and Crisp, 2008), and an assumed generation time of 20 years. This is a somewhat arbitrary value based on the longevity of individuals, the time to maturation and the occurrence of clonal reproduction (via nucellar embryony) in some species (M.L. Roose, pers. obs.).  This value is not critical because we have a reasonable estimate of the time of divergence used to calculate μ and therefore the interaction between generation time and *N*_e_ balances out (as can be deduced using the equations presented in Rosenberg, 2003).

For step 4, θ was estimated using MCMCcoal (Rannala and Yang, 2003; Yang, 2002) following an analysis of 100k generations, with 5k generations discarded as the “burnin” (similar results were obtained after only 10k generations, indicating that this run length is sufficient).  The sequence data were obtained from alleles found in individuals that were considered pure, or that grouped with pure individuals of trifoliate orange, pummelo, citron or mandarin (as found in our phylogenies).  Pure individuals for each species are based on previously published STRUCTURE results (Barkley et al., 2006) and the analysis done by us using the same data and slightly different set of accessions.  Kumquat was excluded because during preliminary analyses we could not reliably identify a clade in each gene that contained the alleles from individuals belonging to this group; also, because of the small size of this group - we included only one kumquat in this study.  The trifoliate orange alleles always formed a monophyletic group, although their sample size was also limited (Figures 1, 2, 3 and Figures S3, S4, S5). The use of pure individuals helped to ensure that *N_e_* was not overestimated by inclusion of alleles introduced by recent hybridization, as is easily detected by STRUCTURE.  Older hybridizations might not be detected by STRUCTURE (if either novel alleles become fully incorporated into a gene pool and/or the novel alleles no longer match their source because of mutations), but can be considered to be part of that gene pool and therefore influence *N_e_*.

MCMCcoal requires a species tree as input file.  We found that the three most resolved genes (HYB, LGT and MDH) had topologies (T,(P,(C,M))) , (T,(P,(C,M))) , and (T,(P,C,M)) respectively, (where T = trifoliate, P = pummelo, C = citron, M = mandarin/sweet orange), once the other (probably hybrid) species were excluded.  P12 had the same topology as MDH, and the cpDNA tree differed only by the placement of citron, but is equivalent to (T,P,C,M) when clades with posterior probabilities below 0.95 are treated as unresolved (Bayer et al., 2009) (as is ATC).  Only CTV11 contradicted this topology of the reduced set of species (with support), but since CTV11 sequences showed recombination, we suspected this gene of being affected by mistaken orthology (see Discussion) and therefore excluded it from consideration here.  We used the input species tree that seemed most likely – namely (T,(P,(C,M))).  However, to test this assumption, we also used the following species trees in MCMCcoal: (T,(C,(P,M))) and (T,(M,(C,P))) to explore the sensitivity of θ to the assumed tree.

The *N_e_* was derived using θ = 4.*N_e_*.μ.  Some *Citrus* cultivars preferentially out-cross or are self-incompatible (Herrero et al., 1996). Hence, the appropriate value to use in this formula to reflect the breeding system is not precisely known.  It will lie somewhere between 2 (for nuclear genes of diploid selfing species, such as citron) and 4 (for obligate out-crossing species, such as pummelo).

For step 5, simulations on the test set chronograms were done assuming a fixed *N_e_* of 8,000 for all species and their common ancestors, with 20 simulated trees produced for each input tree. An *N_e_* of 8,000 is close to the mean of the estimated values across species, ancestral populations and genes (see Results and Table 3).  However, this value might be conservative (favouring the null hypothesis) relative to what we are testing, because it is nearly twice the value in the ancestral populations (c. 4,000 – 5,000) where lineage sorting would have taken place that might otherwise appear similar to hybridization (see Results).  The *N_e_* of mandarin (> 13,000) appears to reflect a population expansion since speciation, thus it will not increase the probability of incongruence due to incomplete lineage sorting among the species.  Inclusion of ancient DNA samples in the study would improve ancestral *N_e_* estimation (Minin et al., 2008). As such samples are not available, a conservative estimate of *N_e_* is necessary.

For step 6, nexus files were made that included the trees to be compared to one another and an analysis block that designated the distance metric to be used (symmetric distance: Penny and Hendy, 1985). Each of the 20 trees drawn from the stable posterior distribution from the BA was placed at the beginning of the block of 20 simulated trees that arose by coalescent simulation. The first tree for each block was then compared to the remainder (using PAUP* commands “treedist metric=symdiff” and “fromtree=1”). All 400 distances generated this way (per locus) were pooled for subsequent steps. The 20 BA trees for each locus were compared pair-wise to one another in a similar manner and also pooled for each pair-wise comparison. The distributions of distances were plotted in Microsoft Excel. For step 7, the null hypothesis of lineage sorting alone was rejected when *any* tree to tree distances for observed gene trees overlap *any* of the null distributions generated by simulation from the observed gene trees at the 80% critical value (see below and Results). We use φ as the test statistic to indicate the degree of overlap, by subtracting the value (of the symmetric distance) of the 80% critical value of the null distribution from the value of the lower credibility interval of the distances among observed gene trees. Only when φ > 0 can the null hypothesis be rejected. The global type 1 error rate of φ has been calculated with this in mind.

Although gene trees may not appear to be useful in generating a null distribution, this was tested by using a known species tree in simulations and then sampling genes and performing the test as though only gene trees were known (Maureira-Butler et al, 2008).  Maureira-Butler et al. (2008) assessed - at what critical value of the null distribution the type 1 error rate of φ was below 5% using two randomly sampled simulated genes across a range of levels of lineage sorting.  We used the same species tree, but added a third simulated gene tree to assess - at what critical value a similar type 1 error rate of φ could be produced. The critical value is the proportion of the null distribution that must fall below the 95% credibility interval of the observed gene tree to tree distances in order to reject the null hypothesis.

This determination of the critical value rests on the assumption that the known species tree we used to test the effectiveness of the test has similar properties to that in *Citrus*. Clearly, the *Citrus* species tree is unknown prior to conducting the test, but it should have these properties in common: (1) a similar (or greater) total tree depth, (2) a similar number of (or fewer) taxa, (3) a similar (or more balanced) shape. The total tree depth and number of taxa taken together give an indication of the average length of branches in the unknown species tree. Branch lengths are the critical parameters in any assessment based on coalescent simulation. Balanced trees tend to spread the branch lengths more evenly, resulting in fewer short branches, on average, than unbalanced trees.

A more difficult property to assess the unknown species tree is the presence of rapid radiation. This is not always diagnosable in gene trees, as these generally will not show similar patterns to the species tree that contains rapid radiations due to the expected time of coalescence. We have assumed, in this case, that radiations are not especially rapid, although acknowledging this assumption is problematic. In summary, our observed gene trees in *Citrus* suggest properties of the unknown species tree. If we simulate using a known species with similar properties, we have a reasonable expectation that the error rates suggested by simulation will hold in the case of *Citrus*.

(1) The depth of the shortest three branches in our test species tree ranged from 2.6 – 0.33, 5.2– 0.65 and 6.5– 0.81 coalescent units, respectively, which includes rather severe conditions. The total tree depth ranged from c. 800k to c. 100k generations. The observed gene trees were calibrated to a crown age of c. 300k generations, based on previous molecular dating, which is overlapped by our simulated conditions. This calibration was derived from a cpDNA gene tree, which is likely to have a younger coalescence time than the nuclear gene trees, on average. This would tend to favour underestimation of the branches in the nuclear gene trees, causing a slight bias in favour of the null hypothesis (incomplete lineage sorting), making the test conservative.

(2) The number of alleles is somewhat less important, where many alleles are grouped in polytomies (any relationship is possible under simulation within a polytomy); rather the number of resolved groups is the better measure of “taxon number” in the observed gene trees. 15 species were included in the test species tree, with a single allele sampled per species. In our observed trees, we detected 15 distinct well-supported allele groups in HYB, seven in LGT and eight in MDH.

(3) The imbalance (Colless’ imbalance) of the observed gene trees was 0.17 (HYB, Figure S1A), 0.2 (LGT, Figure S1B) and 0.19 (MDH, Figure S1C) – rather typical values when compared to trees simulated under the Yule model for this number of taxa. In the test species tree, the imbalance was deliberately inflated (0.42) to provide some buffer against the possible presence of rapid radiation that is difficult to detect from a few gene trees.

Results

We found that at 80% critical value, the type 1 error rate of φ of < 5% was maintained across the range of levels of lineage sorting that we tested (the same as in Maureira-Butler et al., 2008, see their supplementary Figure 3).  We therefore used this critical value when testing the null hypothesis of lineage sorting alone to explain incongruence among HYB, LGT and MDH in *Citrus*.

Twenty trees were drawn from the stable posterior distribution. After alleles were pruned to leave one per individual maximizing incongruence (Figure S1), the trees were converted to chronograms using penalized likelihood (PL) with smoothing parameters of 0.25, 0.5 and 0.004 for HYB, LGT and MDH, respectively, after cross validation. Mutation rates (per year and generation) derived from the chronograms for each locus are reported in Table 3. Theta (θ) was estimated using several species trees and across loci. Median *N_e_* values were derived from θ and mutation rate and ranged from c. 2,000 to 21,000 (Table 3) for the first species tree listed in the Methods, with across loci means of medians between c. 4,000 and 13,000. Importantly, ancestral *N_e_* (c. 4,000 – 5,000) was lower than extant species’ *N_e_* (c. 5,000 – 13,000), the latter indicative of post-speciation population expansions that do not affect the probability of lineage sorting among these species. We chose to err towards acceptance of the null hypothesis by using a value slightly above the mean of all median values (8,000).  Alternative species trees used in MCMCcoal caused *N_e_* estimates to differ by only a few hundred individuals (details not shown). Therefore, the estimate of *N_e_* appears to be robust to error in the assumed species tree.
